# Fibroblast Transcriptomics in Molecular Diagnostics of a Comprehensive Dystonia Cohort

**DOI:** 10.1002/ana.78171

**Published:** 2026-02-02

**Authors:** Alice Saparov, Ivana Dzinovic, Theresa Brunet, Vicente A. Yépez, Florian Hölzlwimmer, Elisabetta Indelicato, Birgit Assmann, Susann Badmann, Diana Ballhausen, Steffen Berweck, Felix Brechtmann, Melanie Brugger, Kevork Derderian, Felix Distelmaier, Philip Harrer, Denisa Harvanova, Petra Havrankova, Ann‐Kathrin Jaroszynski, Miriam Kolnikova, Robert Kopajtich, Anne Koy, Magdalena Krygier, Lukas Kunc, Katarina Kusikova, Oliver Maier, Maria Mazurkiewicz‐Bełdzińska, Christian Mertes, Ava Oberlack, Timo Roser, Alexandra Sitzberger, Ugo Sorrentino, Antonia M. Stehr, Katharina Vill, Matias Wagner, Holger Prokisch, Sylvia Boesch, Jan Necpal, Robert Jech, Juliane Winkelmann, Elisabeth Graf, Julien Gagneur, Matej Skorvanek, Michael Zech

**Affiliations:** ^1^ Institute of Human Genetics, School of Medicine and Health Technical University of Munich Munich Germany; ^2^ Institute of Neurogenomics, Helmholtz Zentrum München Munich Germany; ^3^ Bavarian Genomes Network for Rare Disorders Technical University of Munich Munich Germany; ^4^ Helmholtz Association ‐ Munich School for Data Science (MUDS) Munich Germany; ^5^ School of Computation, Information and Technology Technical University of Munich Garching Germany; ^6^ OmicsDiscoveries GmbH Planegg Germany; ^7^ Center for Rare Movement Disorders Innsbruck, Department of Neurology Medical University of Innsbruck Innsbruck Austria; ^8^ Medical Faculty Heidelberg, Center for Pediatric and Adolescent Medicine, Department I, Division of Pediatric Neurology and Metabolic Medicine Heidelberg University Heidelberg Germany; ^9^ Pediatric Metabolic Unit, Pediatrics, Woman‐Mother‐Child Department Lausanne University Hospital and University of Lausanne Lausanne Switzerland; ^10^ Center of Child Neurology, Developmental Medicine, and Rehabilitation Children's Hospital of Eastern Switzerland St. Gallen Switzerland; ^11^ Departments of General Pediatrics, Neonatology, and Pediatric Cardiology, Medical Faculty and University Hospital Düsseldorf Heinrich‐Heine‐University Düsseldorf Germany; ^12^ Associated Tissue Bank, Faculty of Medicine, P. J. Safarik University and L. Pasteur University Hospital in Kosice Kosice Slovakia; ^13^ Department of Neurology, First Faculty of Medicine and General University Hospital in Prague Charles University Prague Czech Republic; ^14^ Department of Pediatric Neurology, Faculty of Medicine Comenius University, University Hospital Bratislava and National Institute of Children's Diseases Bratislava Slovakia; ^15^ Department of Pediatrics, Faculty of Medicine and University Hospital Cologne University of Cologne Cologne Germany; ^16^ Center for Rare Diseases, Faculty of Medicine and University Hospital Cologne University of Cologne Cologne Germany; ^17^ Department of Developmental Neurology Medical University of Gdansk Gdansk Poland; ^18^ Division of Pediatric Neurology and Developmental Medicine and LMU Center for Children with Medical Complexity, Dr. von Hauner Children's Hospital, LMU Hospital Ludwig‐Maximilians‐Universität Munich Germany; ^19^ Department of Neurology Zvolen Hospital Zvolen Slovakia; ^20^ Parkinsonism and Movement Disorders Treatment Center Zvolen Hospital Zvolen Slovakia; ^21^ Department of Neurology P.J. Safarik University Kosice Slovakia; ^22^ DZPG, Deutsches Zentrum Für Psychische Gesundheit Munich Germany; ^23^ Munich Cluster for Systems Neurology, SyNergy Munich Germany; ^24^ Computational Health Center, Helmholtz Center Munich Neuherberg Germany; ^25^ Department of Clinical Neurosciences P.J. Safarik University Kosice Slovakia; ^26^ Department of Neurology University Hospital of L. Pasteur Kosice Slovakia; ^27^ Institute for Advanced Study Technical University of Munich Garching Germany

## Abstract

**Objective:**

Genomic sequencing leaves >50% of dystonia‐affected individuals without a diagnosis. Where DNA‐oriented approaches remain insufficient, integrating multiomics is essential to advance genome interpretation. Herein, we incorporated RNA sequencing (RNA‐seq) data from 167 patients with dystonia across a range of ages and presentations.

**Methods:**

We leveraged an RNA‐seq analysis pipeline, focused on the identification of expression and splicing aberrations, on RNA‐seq from skin biopsies. The recruited patients had early‐onset dystonia in 85.0%, non‐focal dystonia in 92.2%, and coexisting features in 76.0%. Thirty‐six patient samples with pre‐identified variants (36/167, 21.6%) and 131 samples with no previously prioritized diagnostic candidates from genomic sequencing (131/167, 78.4%) were evaluated.

**Results:**

We found that >80% of dystonia‐associated genes were detected by fibroblast RNA‐seq. Expression and splicing aberration analyses produced a manageable number of significant RNA defects affecting dystonia‐associated genes. The approach was especially successful in validating pathogenic effects of loss‐of‐function variants, with disease‐relevant RNA‐underexpression detected for 66.7% (10/15). Studying aberrant expression and splicing in the context of other pre‐identified variant types yielded relevant results in 28.6% (6/21 samples). We obtained a 6.9% (9/131) diagnostic uplift for patients without prior candidates, all of whom exhibited combined dystonia with autosomal recessive inheritance. The new diagnoses from RNA‐seq and genomic reanalysis were based on previously neglected splice‐region (3/9) and deep(er) intronic (6/9) variants. For the observed events, integration of new machine‐learning scores predicted corresponding aberrant gene expression in the brain.

**Interpretation:**

Fibroblast‐based RNA‐seq in our selected cohort improved variant interpretation and offered a modest yield in patients without prior candidate variants. ANN NEUROL 2026;99:1363–1378

Although variants in over 300 genes are linked to dystonias, diagnostic difficulty and misdiagnoses are still common.^1^ Intending to resolve a range of undiagnosed presentations of dystonia, we previously created a collaborative research infrastructure that enables application of emerging innovative approaches for etiological discovery.^2^, ^3^ Whole‐genome sequencing (WGS) has recently been performed in addition to previous whole‐exome sequencing (WES) on difficult‐to‐diagnose cases from our cohort, but only achieved an added diagnostic yield of 12%.^3^ A substantial percentage of WGS variants carried by our study participants were uninterpretable due to indeterminate consequences, resulting in a lack of reporting or prioritization.^3^ RNA sequencing (RNA‐seq) is a powerful complementary assay for investigating the coding and non‐coding genome, allowing for transcriptome‐wide detection of aberrant gene expression and defective splicing as evidence for variant pathogenicity as well as specific pathophysiological outcomes.^4^, ^5^, ^6^ A few disease scenarios demonstrated the value of RNA‐seq for the evaluation of transcriptional perturbations underlying monogenic conditions based on skin biopsies,^7^, ^8^, ^9^, ^10^, ^11^, ^12^ an easily obtainable source material.^5^, ^7^ However, specific tissue expression profiles of dystonia‐linked genes suitable for clinical RNA‐seq‐based diagnostics have not been thoroughly delineated. Moreover, whereas RNA‐seq has added critical insights for prioritizing pathogenic variants in individual dystonia subjects,^3^ systematic diagnostic utilization of this method in a larger patient group has not yet been realized.

Here, we applied an automated RNA‐seq analysis pipeline^13^ to fibroblasts from 167 unrelated dystonia‐affected individuals, the largest compendium collected in this indication to date.

## Methods

### Dystonia Fibroblast Collection

**FIGURE 1:**
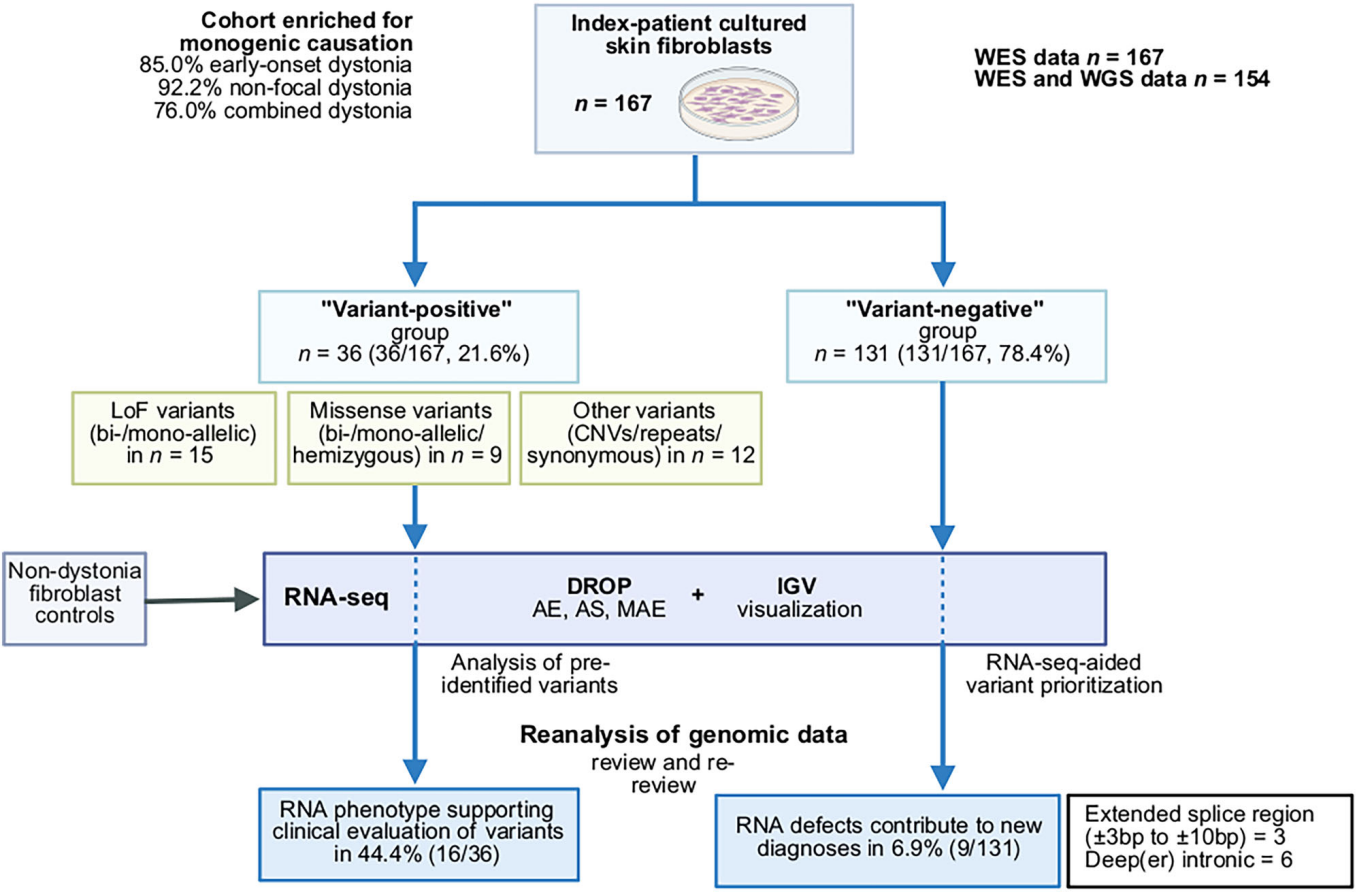
Overview of the RNA‐seq pipeline for fibroblasts from dystonia‐affected individuals. All recruited skin fibroblasts from patients with pre‐identified variants (“variant‐positive” group, 21.6%) and patients for whom WES/WGS alone had not yielded a relevant diagnostic variant candidate (“variant‐negative” group, 78.4%) received RNA‐seq. The data were analyzed using the DROP workflow,^13^ direct visualization in IGV,^19^ and comprehensive genomic re‐evaluation. We assessed the outcomes of RNA‐seq for both patient groups, considering the range of aberrant RNA phenotypes resulting from various variant categories, the benefits in aiding clinical interpretation, and the added diagnostic yield. We note that the reanalysis of genomic profiles of patients in the context of fibroblast RNA‐seq results may benefit in the (near) future from additional solutions for even more accurate genomic variant evaluation such as long‐read genome sequencing. AE = aberrant expression; AS = aberrant splicing; CNVs = copy‐number variants; DROP = Detection of RNA Outliers Pipeline; IGV = Integrative Genomics Viewer; LoF = loss‐of‐function; MAE = mono‐allelic expression; RNA‐seq = RNA sequencing; WES = whole‐exome sequencing; WGS = whole‐genome sequencing. [Color figure can be viewed at www.annalsofneurology.org]

Using our multicenter network,^2^, ^3^ fibroblast samples were collected from 167 index patients (77 female patients, 46.1%, mean age at sampling = 21.0 years, range = 1–69 years) who were clinically diagnosed with rare dystonic syndromes^14^ (Fig 1). Between 2021 and 2024, these patients underwent standard punch biopsies for skin‐specimen acquisition by collaborators in tertiary movement‐disorders centers with focus on both pediatric and adult forms of dystonia in Austria, Czechia, Germany, Poland, Slovakia, and Switzerland; 95.2% of index patients (159/167) were of European background. Informed written consent was obtained, the institutional ethics review boards of the collaborating partners approved the procedures (main reference numbers = 208/21S and 589/20S), and the study was conducted in accordance with the principles of the Declaration of Helsinki. The set of enrolled individuals had extensive prior genomic investigations, including WES‐only (13 cases with likely pathogenic/pathogenic^15^ or candidate variants from this analysis), and WES with follow‐up WGS studies (n = 154).^2^, ^3^ We favored both cases with pre‐existing findings from WES/WGS (“variant‐positive” group) and cases without prioritized variants but with suspicion of an underlying genetic disorder^3^, ^16^ (“variant‐negative” group) for fibroblast‐culture establishment and RNA‐seq (see Fig 1): pre‐identified variants of interest, often affecting newly discovered or less‐established dystonia‐related genes, were present in 36 individuals (21.6%); in 14 of the subjects (14/36, 38.9%), results from RNA‐seq provided additional support for variant pathogenicity; in 2 other cases (2/36, 5.6%), RNA evidence‐supported reclassification of variants^15^, ^17^ was enabled, leading to new diagnoses (see Fig 1, see Results and Supplementary Table S1). The remaining 131 patients (78.4%) lacked a selected variant candidate and were enrolled for RNA‐seq data‐aided genomic reanalysis.^10^, ^18^ The majority of patients with sampled fibroblasts displayed early‐onset (85.0%), non‐focal (92.2%), and/or combined dystonia (76.0%); a summary is given in Figure 1. The control fibroblast‐based RNA‐seq cohort included samples from 182 unrelated research donors who had been referred to our sequencing platform for various clinical indications except dystonia.

### RNA‐Sequencing

Between 2022 and 2025, RNA sequencing and processing for dystonia cases and controls were performed according to standard methodologies, integrating established equipment and reagents, protocols, and quality‐control parameters (RNA integrity number (RIN) > 7).^3^, ^7^, ^10^ The mean turnaround time from skin‐biopsy acquisition to RNA‐seq for dystonia cases was 11 months (range = 3–16 months), and all included fibroblasts (mean passage number = 5) had been cultured according to standard conditions^3^ and tested negative for mycoplasma contamination. RNA libraries were created using Illumina Stranded mRNA Prep Ligation, polyA‐tailed kits. A NovaSeq6000 instrument (Illumina) was used to produce 100‐bp paired‐end reads with a mean number of 76.7 million (range = 44.9–146.6 million) read pairs per sample; with a minimal read‐depth acceptance of 30 million paired reads, no sample failed the required cutoff value for quality. STAR version 2.4.2a was deployed to align reads to the GRCh37/hg19 reference; on average, 85.7% of uniquely mapped reads were achieved for all samples. The processed alignment files were used for visualization of genomic variant‐related RNA defects in the Integrative Genomics Viewer (IGV),^19^ and as input for parallelized analysis of aberrant events in the Detection of RNA Outliers Pipeline (DROP version 1.4.0, https://github.com/gagneurlab/drop),^13^ comprising our standardized framework for transcriptome‐wide studies. In DROP, batch effects are mitigated within the individual DROP modules through a denoising autoencoder framework which learns latent factors to control for covariation.^13^

### RNA Expression Profiles and Disease‐Gene Lists

“Expressed genes” were defined in DROP as genes for which >5% of the analyzed samples exhibited fragments per kilobase of transcript per million mapped reads (FPKM) values of >1.^13^, ^18^ These expressed genes were filtered with disease‐gene lists, obtained as follows: (i) Online Mendelian Inheritance in Man (OMIM; accessed October 15, 2024)^20^ and published compendia (accessed July 30, 2025)^21^ were queried for human morbid genes^20^ and genes associated with dystonia^20^ or “rare neurological disease”^21^; (ii) MDSGene^22^ and GeneReviews^23^ were accessed (May 9, 2025) to extract curated descriptions of isolated dystonia‐related genes; and (iii) dystonia‐pathway genes were manually reviewed (up to May 9, 2025) as an expansion of a compendium recently compiled by us.^24^ To compare the results from RNA‐expression profiling in fibroblasts to a second clinically accessible tissue, we also examined the numbers of expressed dystonia‐related genes in 279 whole‐blood RNA‐seq samples that were available to us from a different research project.

### Global RNA‐Defect Studies

Evaluating outlier status for expression, splicing, and allele imbalance in RNA‐seq data involved previously reported procedures,^13^ streamlined on our locally installed bioinformatics infrastructure.^10^ We used DROP^13^ version 1.4.0, incorporating Outlier in RNA‐Seq Finder (OUTRIDER)^25^ version 1.20.1 for identification of aberrant expression (AE) and Find RAre Splicing Events in RNA‐seq (FRASER) 2.0^26^ version 1.99.4 for discovery of aberrant splicing (AS). A false‐discovery rate (FDR) threshold of ≤0.05 after multiple‐testing correction was used for significant AE outcomes, whereas an FDR of ≤0.05 with an effect size |*Δ*J| ≥0.1 was required for significant AS events. We also ran a negative binomial test‐based tool for the detection of mono‐allelic expression (MAE).^7^, ^13^ Subsequently, expression abnormality‐associated *z*‐score and fold‐change values, as well as splice‐defect metrics and MAE hits were explored at an individual patient basis. Considering the expected rarity of individual causative molecular lesions, our analytic strategies were based on the comparison of one case subject against all the remaining samples (patients with dystonia and controls, n = 349).^10^

### Association of Pre‐Identified Variants with RNA Phenotypes

For each patient from the “variant‐positive” group, we assessed AE, AS, and MAE in the context of previous genomic findings. Genes and variants of interest were associated with the corresponding outcomes from DROP.^10^ Pre‐identified variants in genes not expressed in fibroblasts were flagged as unanalyzable (see Supplementary Table S1). The pre‐identified variants were further manually investigated for possible effects on splicing patterns or associated allele‐biased expression using simultaneous visualization of RNA‐seq and WES/WGS data in IGV. We considered observed AE and/or AS events as supportive for variant pathogenicity if the gene carrying a pre‐identified likely pathogenic/pathogenic variant was affected by a corresponding RNA defect. New diagnoses were considered if RNA evidence‐supported reclassification of variants^15^, ^17^ was enabled.

### Reanalysis of WES/WGS Variants Aided by RNA‐Seq Data

DROP results for AE, AS, and MAE were used in the case‐by‐case reanalysis of WES/WGS data in the “variant‐negative” group.^10^ For clinical evaluation, we narrowed the transcriptome‐wide outlier summary files down to significant hits (FDR ≤ 0.05) that were associated with monogenic disorders in OMIM and specifically focused on genes linked to dystonia and related neurological conditions consistent with the observed phenotypic presentations.^20^ MAE analysis was restricted to hits associated with rare variants (minor‐allele frequency = < 0.001 in gnomAD and <0.05 in dystonia‐patient and control datasets; allelic imbalance = >85%). Informed by the DROP‐output shortlists, we revisited variants affecting plausible genes in the individual patients. Results from complementary tests were taken into account when possible, including findings from additional segregation, proteomics^3^ (see the Table 1 and Supplementary Fig S1), long‐read sequencing (Supplementary Fig S2), laboratory (Supplementary Fig S3), and reverse‐phenotyping analyses; results from orthogonal validations and confirmatory evaluations are summarized in Supplementary Table S2. Given that we opted for diagnostic‐grade procedures of RNA‐seq implementation, we expect that the herein‐described variant‐prioritization strategies and reporting thresholds would not relevantly differ in the context of clinical applications. It should, however, be noted that in a real‐world scenario more comprehensive optimizations of RNA‐seq‐guided genomic reanalyses approaches may be required, like relaxed filtering for borderline nonsignificant expression and splicing alterations in an expanded set of candidate genes.

**TABLE 1:** Index Patients With New Diagnostic Findings After Genomic Reanalysis Aided by RNA‐Seq

Index patient/age/sex	Phenotype	Gene/diagnosis (OMIM)	Variant(s)	Variant type/zygosity	Detected significant outlier event(s); RNA phenotype
Extended splice‐region variants (±3 bp to ± 10 bp region)					
R030/44 yr/M	Dystonia, spasticity, ataxia	*ACP33* (*SPG21*) Mast syndrome (248900)	NM_016630.7: c.306 + 6 T>A	Intronic SNV/hom	*ACP33* (*SPG21*): AS; exon‐4 skipping^a^
R054/22 yr/F	Dystonia, ataxia, ID, gingival hyperplasia, cerebellar hypoplasia	*ATG7* Spinocerebellar ataxia‐31 (619422)	NM_001349232.2: c.528 + 3A>G/c.1090G>A, p.Gly364Ser	Intronic SNV/missense SNV/comp het	*ATG7*: AS; exon‐8 skipping
R089/8 yr/F	Dystonia, DD, epilepsy	*GLS* Developmental and epileptic encephalopathy‐71 (618328)	NM_014905.5: c.1713‐9C>G	Intronic SNV/hom	*GLS*: AS; exon‐16 extension, skipping of exons 16 and 17^a^
Deep(er) Intronic Variants (> ± 10 bp From Exon‐Intron Boundaries)					
R082/8 yr/M	Dystonia, chorea, ID	*SHQ1* Neurodevelopmental disorder with dystonia and seizures (619922)	NM_018130.3: c.144‐24A>G/ c.487G>T, p.Asp163Tyr	Intronic SNV/missense SNV/comp het	*SHQ1*: AE, AS; significant RNA underexpression (FC = 0.51), exon‐2 extension (c.144‐24A>G), exon‐5 skipping (c.487G>T)
R140/12 yr/F	Dystonia, stereotypies, DD, cerebellar atrophy	*SNX14* Spinocerebellar ataxia‐20 (616354)	NM_153816.6: c.1108 + 28A>G	Intronic SNV/hom	*SNX14*: AE, AS; significant RNA underexpression (FC = 0.23), exon‐12 extension
R158/2 yr/M	Dystonia, DD, eye movement abnormalities	*AGTPBP1* Neurodegeneration, childhood‐onset, with cerebellar atrophy (618276)	NM_001330701.2: c.1303‐4238C>G	Intronic SNV/hom	*AGTPBP1*: AE, AS; significant RNA underexpression (FC = 0.20), pseudoexon splicing‐in^a^
R134/23 yr/F^b^, ^c^	Dystonia, DD (during childhood)	*ATM* Ataxia‐telangiectasia (208900)	NM_000051.3: c. 3284 + 695G>T; c.3284 + 699A>C	Intronic MNV/hom	*ATM*: AE, AS; significant RNA underexpression (FC = 0.38), pseudoexon splicing‐in^a^
R105/25 yr/M^b^	Dystonia, spasticity	*SPG11* Spastic paraplegia‐11 (604360)	NM_025137.4: c.1235C>G, p.Ser412*/ c.3454‐28A>G	Nonsense SNV/intronic SNV/comp het	*SPG11*: AE, AS; significant RNA underexpression (FC = 0.22), exon‐20 skipping, partial retention of introns 19 and 20^a^
R058/16 yr/M^b^	Dystonia, spasticity, DD	*UFC1* Neurodevelopmental disorder with spasticity and poor growth (618076)	NM_016406.4: c.244_255del, p.Glu83_Ile86del/ c.255 + 17G>A	Inframe indel/intronic SNV/comp het	*UFC1*: AE, AS; significant RNA underexpression (FC = 0.59), exon‐3 skipping^a^

^a^ Significant underexpression of the encoded protein detected on proteomics analysis (proteomics data available for fibroblast samples from patients R030, R089, R158, R134, R105, and R058).

^b^ Index patient and causative intronic variants previously reported in detail in a multi‐omics companion manuscript (PMID: 39937650).

^c^ Index patient R134 had no prior clinical suspicion of a diagnosis of ataxia‐telangiectasia; following genomic re‐analysis and re‐prioritization of *ATM* variants, alpha‐fetoprotein was measured in blood and found to be present in pathologically raised levels.

AE = aberrant expression; AS = aberrant splicing; bp = base pairs; comp het = compound heterozygous; DD = developmental delay; F = female; FC = fold change; hom = homozygous; ID = intellectual disability; M = male; MNV = multi‐nucleotide variation; OMIM = Online Mendelian Inheritance in Man; RNA‐seq = RNA sequencing; SNV = single‐nucleotide variant; yr = years.

### Prediction of Variant‐Related Aberrant RNA Expression in the Brain

We modeled the impact of patient‐specific genetic perturbations on RNA expression in dystonia‐relevant brain regions using AbExp.^27^ Identified variants that were associated with corresponding significant gene‐underexpression outliers in fibroblasts were included in the analysis; for details, see Supplementary Figures S4 and S5.

## Results

### Dystonia‐Related Genes Covered by Fibroblast‐Based RNA‐Seq

**FIGURE 2:**
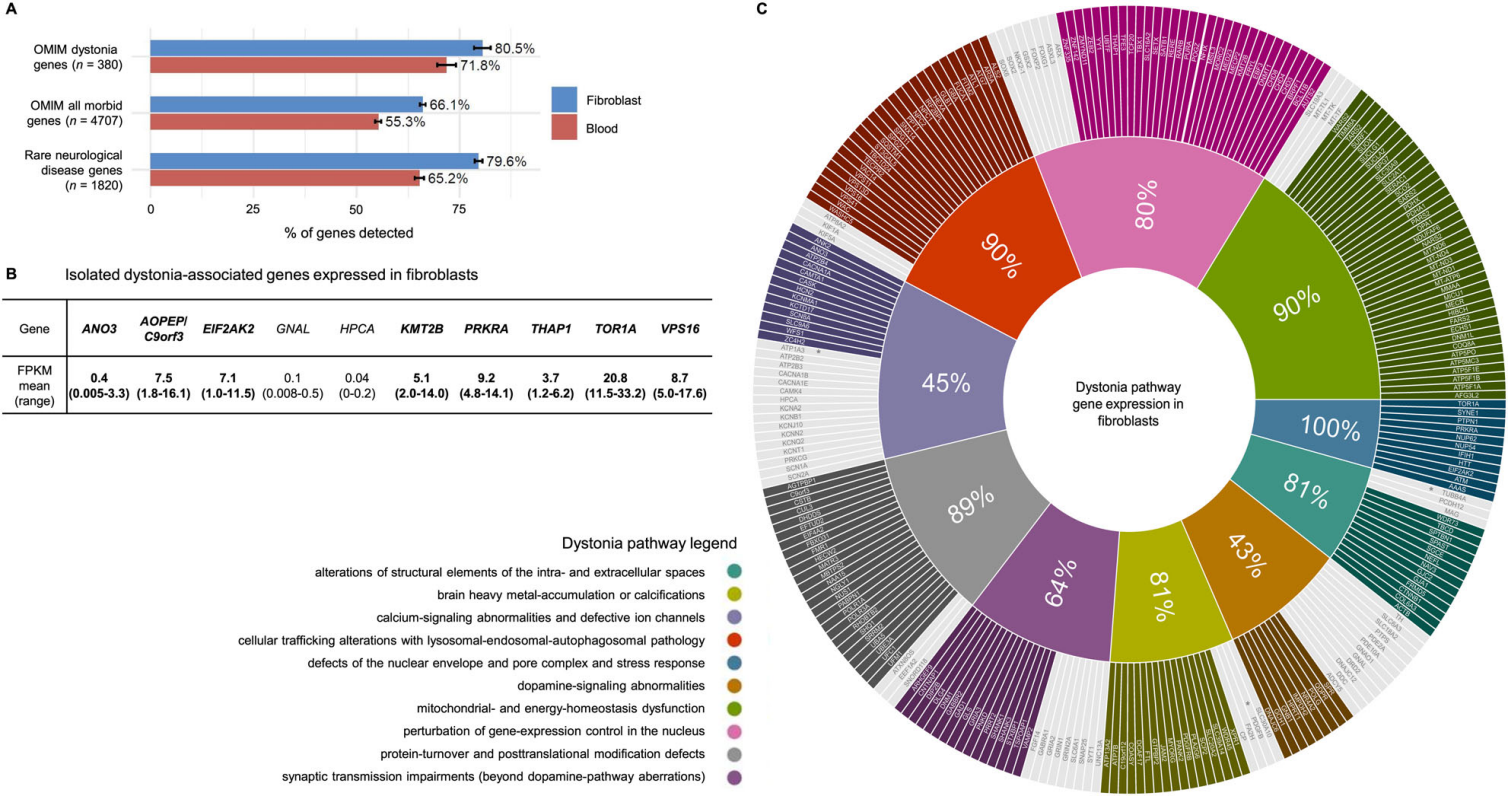
Detection of dystonia‐associated gene expression by RNA‐seq. (A) Percentages of different gene sets that were covered by RNA‐seq, as defined by the DROP workflow,^13^ in fibroblasts from our patients and controls (n = 349). Dystonia‐associated genes (n = 380) and all human morbid genes (n = 4,707) were obtained by systematic OMIM queries,^20^ as described.^2^, ^3^ The “rare neurological disease gene” set (n = 1,820) has been published recently.^21^ Corresponding gene coverages in blood samples are provided for comparison. For each percentage of detectable genes, errors bars represent standard errors of the mean. (B) Summary of curated isolated dystonia‐associated genes^22^, ^23^ and their detectable expression in all fibroblast samples included in this study. All genes except for 2 (*HPCA* and *GNAL*, not shown in bold) reached the “expressed gene” threshold.^13^, ^18^ FPKM scores (mean, ranges) in our 349 total samples are given for each gene. (C) Dystonia‐associated gene coverage by RNA‐seq according to 10 reported disease‐relevant molecular pathways.^24^ Percentages of covered genes are highlighted. The pathway genes were manually curated based on previous reviews^1^, ^24^ and continuous searches of publicly available data,^20^ but the lists cannot be regarded as exhaustive complete collections and it is possible that other relevant genes were not added. Despite this limitation, the given percentages are likely representative of the approximate fractions of genes that are covered by RNA‐seq in the different pathways. Genes expressed in blood and not in fibroblasts are marked by asterisks. Color code is provided for the pathways. DROP = Detection of RNA Outliers Pipeline; FPKM = fragments per kilobase of transcript per million mapped reads; OMIM = Online Mendelian Inheritance in Man; RNA‐seq = RNA sequencing. [Color figure can be viewed at www.annalsofneurology.org]

Consistent with previous results,^10^, ^11^ an average of 15,180 total genes were detected through RNA‐seq in patient fibroblasts. Overall, the fibroblasts expressed 80.5% (306/380) of genes linked to dystonic disorders in OMIM^20^ (Fig 2A). This corresponded to a higher percentage of testable genes compared with the expression of all OMIM‐morbid genes (66.1%, 3109/4707),^20^ and a similar rate of gene coverage compared with a recently published “rare neurological disease gene” set (79.6%, 1448/1820)^21^ (see Fig 2A). When focusing on genes primarily involved in isolated dystonia according to reference databases (n = 10),^22^, ^23^ we observed consistently high coverage (80%); only *GNAL* and *HPCA* did not reach the expressed gene threshold (Fig 2B). Gene‐informed molecular pathways are increasingly recognized in dystonia.^1^ Thus, we investigated what fractions of genes within biologically defined pathways for dystonias^24^ could be analyzed by RNA‐seq. Our fibroblast‐based approach detected expression of all genes in 1 of 10 pathways, whereas measurable dystonia‐related gene expression profiles between 43% and 90% were found for the remaining 9 pathways (Fig 2C). Most pathways showed detectable gene representation levels between 60% and 80% (see Fig 2C). As the discovery of gene underexpression caused by loss‐of‐function (LoF) variants was a major contributor to diagnostic success in previous RNA‐seq studies,^8^, ^9^, ^10^ we also explored how many dystonia‐related pathway genes were associated with LoF mechanisms; of those pathway genes expressed in fibroblasts, approximately 82.1% (165/201) were implicated in haploinsufficiency, X‐linked, or recessive disorders with reported causative null alleles,^20^, ^28^, ^29^ indicating that fibroblasts represented a suitable tissue for RNA‐seq‐based evaluation of a variety of dystonia‐related genes that participate in key cellular disease processes. Finally, comparison of gene expression between blood and fibroblasts highlighted that the latter provided a more comprehensive transcriptional landscape for successful application of RNA‐seq‐based dystonia diagnostics (71.8%, 273/380, of dystonia‐related genes from OMIM expressed in blood; see Fig 2A); only 7 dystonia‐related genes from OMIM (*ATP1A3*, *NTNG2*, *PDGFB*, *RNASEH2B*, *SNCA*, *TPK1*, and *TUBB4A*) were expressed in blood but not in fibroblasts.

### Aberrant Events Detected by Automated RNA‐Seq Data Analysis

**FIGURE 3:**
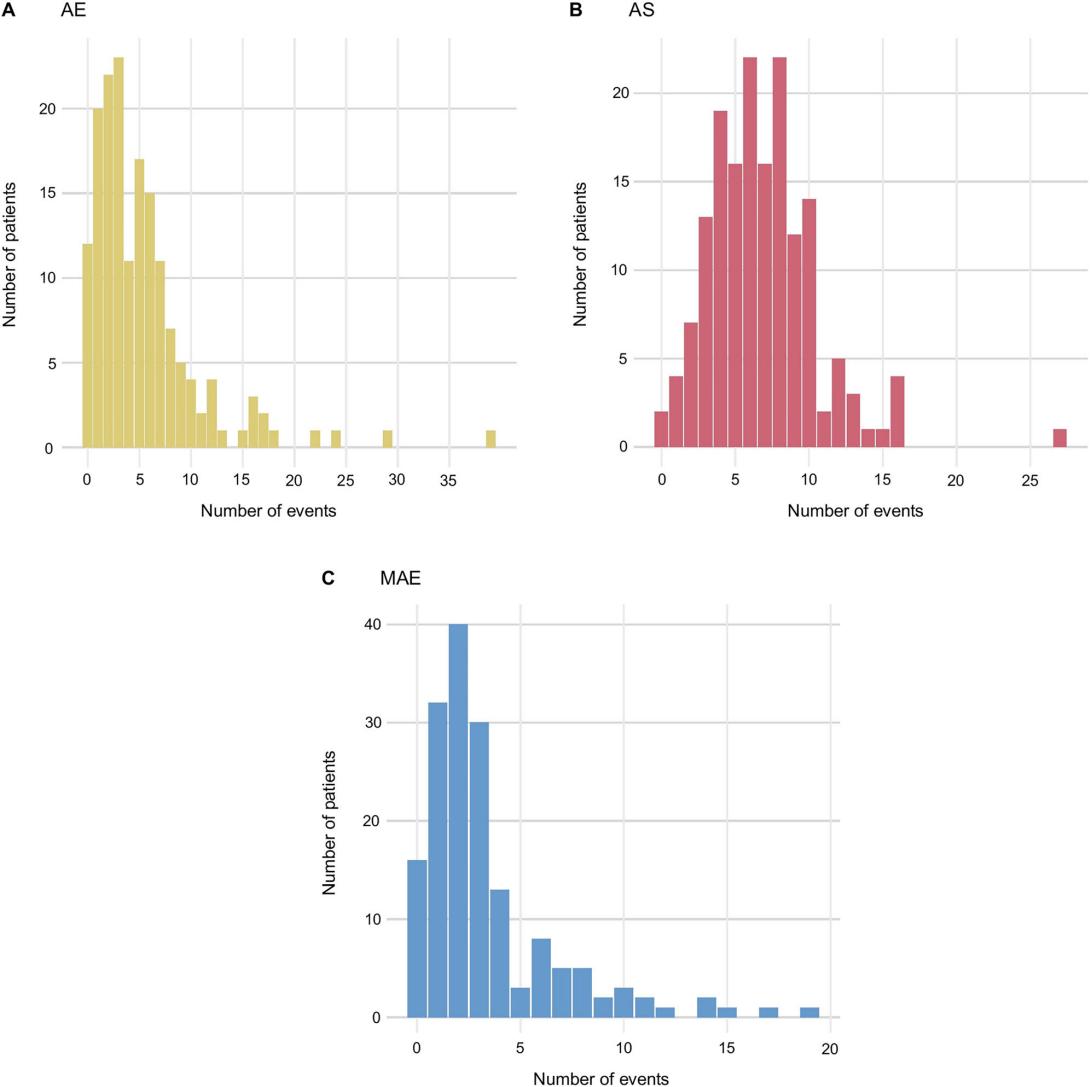
Summary of significant outlier events identified by automated bioinformatic analyses in dystonia RNA‐seq samples. We applied the DROP workflow^13^ to the RNA‐seq data from our 167 dystonia‐affected individuals, allowing for systematic identification of AE, AS, and MAE events through the OUTRIDER,^25^ FRASER 2.0,^26^ and allele‐biased expression discovery^7^, ^10^ modules, respectively. Distributions of detected significant AE hits (FDR ≤ 0.05; n = 882 total events) (A), significant AS hits (FDR ≤ 0.05, n = 1,122 total events) (B), and significant MAE hits (FDR ≤ 0.05, n = 553 total events) (C) for the patient cohort are shown. Only rare genomic variants (MAF < 0.001 in gnomAD and <0.05 in the present patient and control cohort) were considered in the MAE analysis (allelic imbalance >85%). AE = aberrant expression; AS = aberrant splicing; FDR = false‐discovery rate; FRASER = Find RAre Splicing Events in RNA‐seq; MAF = minor allele frequency; MAE = mono‐allelic expression; OUTRIDER = Outlier in RNA‐Seq Finder; RNA‐seq = RNA sequencing. [Color figure can be viewed at www.annalsofneurology.org]

OUTRIDER uncovered on average 5 significant expression outliers (range = 0–39) per patient (Fig 3A). Underexpression was more commonly observed, with 72.9% (643/882) of all significant outliers in patients demonstrating fold changes (FCs) between 0.01 and 0.84. In the FRASER 2.0 analysis with standard settings, each patient sample typically presented 7 splicing outliers (mean; range = 0–27; Fig 3B). The mean number of rare variants with significant allelic imbalance per sample was 3 (range = 0–19; Fig 3C). When refining the analysis by only considering dystonia‐related genes according to OMIM,^20^ a range of 0 to 2, 0 to 3, and 0 to 1 hits were identified in each of the patients by OUTRIDER, FRASER 2.0, and the MAE‐detection module, respectively.

### RNA Abnormalities in Patients with Pre‐Identified Variants

**FIGURE 4:**
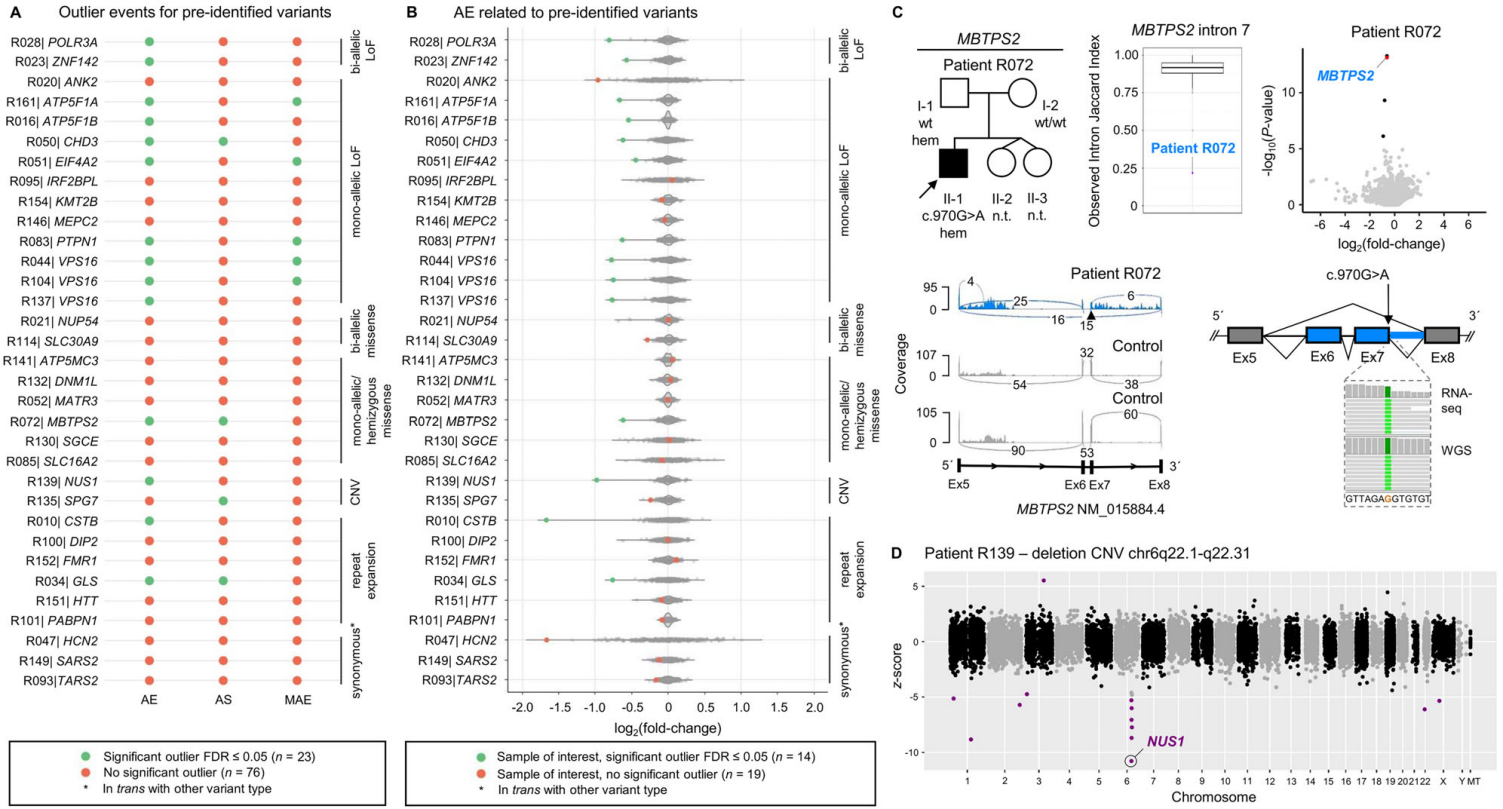
RNA phenotypes associated with pre‐identified variants and examples of readouts aiding in clinical interpretation. (A) Summary of DROP‐workflow^13^ outcomes for all pre‐identified variants that were expressed in fibroblasts and assessed by RNA‐seq in this study. The colored dots indicate whether or not a given WES/WGS‐identified variant produced a significant outlier hit in the AE, AS, and/or MAE analyses. Color code is provided. All subjects with repeat expansion findings had predominant dystonia manifestations, and the here‐reported repeat expansions were interpreted as disease‐related. Samples with variants in *ADCY5*, *ATP2B2*, and *ATXN8OS/SCA8*, unexpressed in fibroblasts, are not shown. (B) Depiction of log2fold changes of RNA expression of the gene of interest for each sample with a pre‐identified variant (*red* or *green dots*) compared to all other samples in which the gene was expressed. Data are based on the output from OUTRIDER,^25^ and each dot indicates a sample. Gene‐wise outlier events representing statistically significant RNA underexpression (AE with FDR ≤ 0.05) are shown in green; color code is provided. All subjects with repeat expansion findings had predominant dystonia manifestations, and the here‐reported repeat expansions were interpreted as disease‐related. Samples with variants in *ADCY5*, *ATP2B2*, and *ATXN8OS/SCA8*, unexpressed in fibroblasts, are not shown. A nonsignificant hit (FDR = 1, *z*‐score = −3.3) in the expression analysis was observed for the CNV sample with *SPG7* homozygous single‐exon deletion, along with another nonsignificant signal (FDR = 0.1, *z*‐score = −4.7) in a sample without identified rare biallelic *SPG7* variation. (C) RNA‐seq‐aided diagnosis of *MBTPS2*‐related IFAP/BRESHECK syndrome in patient R072, highlighted by an arrow in the pedigree. OUTRIDER^25^ and FRASER 2.0^26^ identified significant outlier events for *MBTPS2*, as represented in the RNA expression volcano plot (underexpression outlier for *MBTPS2* highlighted) and the splice metric rank box plot^26^ (mis‐splicing outlier in R072 highlighted). The AE and AS events resulted from a de novo hemizygous c.970G>A predicted missense variant, previously classified as a VUS.^15^ RNA‐seq sashimi plot shows skipping of exons 6 and 7 as well as retention of intron 7 as consequences of c.970G>A in R072 (*blue*), not observed in representative control fibroblasts (*gray*). The location of the *MBTPS2* variant is indicated by a triangle. In the schematic representation of the mis‐splicing event, IGV^19^ screenshots of visualized WGS and RNA‐seq data depict the read pileups at the mutant chromosomal position. (D) RNA‐seq Manhattan plot representation of a 6q22.1‐q22.31 microdeletion involving the dystonia‐linked gene *NUS1* in patient R139. OUTRIDER^25^ identified significant AE (FCs = 0.44–0.59) of 6 genes in the affected chromosomal region 6q22.1‐q22.31 (*purple dots*), including *NUS1* (FC = 0.51), supporting the presence of the pathogenic CNV in the patient. The X‐axis represents chromosomes, and the Y‐axis represents z‐scores. The specific FDR and z‐scores of the affected 6 genes were: *ASF1A*, 8.3 × 10^−11^, −8.69; *CEP85L*, 3.81 × 10^−5^, −7.06; *GOPC*, 0.014, −5.3; *MAN1A1*, 0.001, −6.01; *NUS1*, 4.8 × 10^−18^, −10.78; and *TBC1D32*, 1.6 × 10^−6^, −7.74. The other CNV investigated in this study involved *SPG7* and was detected as a significant hit in FRASER 2.0: FDR = 2.3 × 10^−25^, called as splicing defect (exon skipping) as a result of a homozygous single‐exon deletion (see also Supplementary Table S1). AE = aberrant expression; AS = aberrant splicing; CNV = copy‐number variant; DROP = Detection of RNA Outliers Pipeline; Ex = exon; FDR = false‐discovery rate; FRASER = Find RAre Splicing Events in RNA‐seq; hem = hemizygous; IGV = Integrative Genomics Viewer; LoF = loss‐of‐function; MAE = mono‐allelic expression; MAF = minor allele frequency; n.t. = not tested; OUTRIDER = Outlier in RNA‐Seq Finder; RNA‐seq = RNA sequencing; VUS = variant of uncertain significance; WES = whole‐exome sequencing; WGS = whole‐genome sequencing; wt = wild type. [Color figure can be viewed at www.annalsofneurology.org]

Of the 36 “variant‐positive” samples included in this evaluation (27 [75.0%] of which were previously published in the frame of single‐gene or larger cohort studies^3^, ^30^, ^31^, ^32^, ^33^, ^34^, ^35^; see Supplementary Table S1), 15 carried bi‐allelic (n = 2) or mono‐allelic (n = 13) likely pathogenic/pathogenic^15^ LoF variants; 9 had bi‐allelic (n = 2) or mono‐allelic/hemizygous (n = 7) missense variants (likely pathogenic/pathogenic^15^ variants in 7 samples; variants of uncertain significance [VUS]^15^ in 2 samples); and 12 were known to harbor other mutation types, either classified as likely pathogenic/pathogenic^15^, ^36^, ^37^ alterations (9 samples) or as VUS^15^, ^36^ (3 samples), including copy‐number variants (CNVs), repeat expansions, and synonymous variants (see Supplementary Table S1). Examining samples with LoF changes showed that perturbation of both alleles resulted in significant gene underexpression (*POLR3A*, FC = 0.57 and *ZNF142*, FC = 0.67; Fig 4A, B, see Supplementary Table S1), whereas the mono‐allelic LoF variants had variable outcomes from our RNA‐seq analysis (see Fig 4A, B, and Supplementary Table S1). In 8 of 13 (61.5%) of the cases with heterozygous LoF variants, we detected significantly decreased expression of the variant‐bearing genes, demonstrating that RNA‐seq can readily aid in identifying gene‐inactivating alleles and in supporting haploinsufficiency as a pathomechanism (see Fig 4A, B, and Supplementary Table S1). OUTRIDER successfully revealed significant AE of *VPS16* (FCs = 0.58–0.59; see Fig 4A, B, and Supplementary Table S1) in 3 unrelated cases with previously diagnosed *VPS16*‐related dystonia,^3^, ^30^ a known common form of autosomal dominant isolated dystonia.^1^ Clinical significance assessment of LoF variants was also improved in the context of newly described disease genes and novel gene‐phenotype correlations. For example, interrogation of AE outliers in patient R083 carrying a heterozygous nonsense substitution in *PTPN1*, a gene that has recently been implicated in dystonic encephalopathy,^31^ confirmed significant reduction of *PTPN1* RNA levels (FC = 0.65) and thus the haploinsufficiency effect of the variant (see Fig 4A, B, and Supplementary Table S1). Although OUTRIDER did not detect corresponding significant outliers in RNA‐seq data for the remaining likely pathogenic/pathogenic heterozygous LoF alterations (see Fig 4A, B, and Supplementary Table S1), closer analysis of the results using cohort‐wide rank‐based methods^3^ showed that one variant (frameshift variant in *ANK2* ^3^) was associated with the lowest expression of the affected gene in comparison to all other samples (sample rank = 1/349, FC = 0.51; OUTRIDER FDR > 0.05; see Fig. 4B). This emphasized the value of complementary analysis strategies in large RNA‐seq datasets to characterize different effects of LoF variants on expression. In line with earlier findings,^7^, ^10^ for most pre‐identified bi‐allelic and mono‐allelic/hemizygous missense variants, we observed no significant RNA outlier events (8/9, 88.9%; see Fig. 4A, B and Supplementary Table S1). However, when present, as seen in patient R072 (Fig. 4C and see Supplementary Table S1), RNA‐seq prompted reconsideration of the significance of a predicted missense variant. Trio‐WES yielded a de novo hemizygous c.970G>A (p.Ala324Thr) substitution in *MBTPS2*, initially classified as a VUS attributed to R072's presentation, consisting of dystonia, developmental delay, and epilepsy, which did not match the typical phenotype of *MBTPS2*‐related IFAP/BRESHECK syndrome^20^; RNA‐seq uncovered a *MBTPS2* splicing defect (exon skipping and intron retention) along with significant *MBTPS2* underexpression (FC = 0.65) associated with the variant, thereby suggesting that *MBTPS2* deficiency may well contribute to this patient's clinical outcome. For 2 pre‐identified CNV calls in affected individuals, our RNA analysis provided supporting evidence for these variations (see Fig. 4A, B and Supplementary Table S1), directly identifying dystonia‐associated structural variants that can be challenging to detect by DNA‐sequencing workflows alone.^38^ One example was found in patient R139, where a heterozygous 6q22.1‐q22.31 microdeletion encompassing *NUS1* ^35^ led to approximately half of the normal expression of 6 adjacent genes located within the CNV (significant outliers in OUTRIDER, FCs = 0.44–0.59; Fig. 4D and see Supplementary Table S1). Our pipeline further allowed for the detection of significant AE of genes affected by autosomal‐recessively inherited repeat expansions,^3^ including *CSTB* (FC = 0.31) and *GLS* (FC = 0.59), whereas none of the 5 pre‐identified repeat expansions associated with autosomal dominant/X‐linked inheritance produced a detectable RNA defect. Finally, for 3 cases with synonymous VUS, each observed in trans with another variant type (see Fig. 4A, B, and Supplementary Table S1), no statistically significant AE status of the affected genes or resultant AS or MAE calls were found by our automated pipeline. However, lowered expression of *HCN2* was evident (sample rank = 1/349, FC = 0.32, OUTRIDER FDR > 0.05; see Fig. 4B) in association with a heterozygous, predicted synonymous c.1560C>T substitution and a heterozygous exon‐6 deletion in patient R047 with dystonia and epileptic encephalopathy (see Supplementary Table S1). Visualized inspection of RNA‐seq data indicated that c.1560C>T created a cryptic splice donor, leading to exon‐5 truncation (data not shown). Although the splicing defect was only evident in a few RNA reads, likely because of nonsense‐mediated decay (NMD),^11^ the finding indicated the disruptive nature of c.1560C>T and established the diagnosis of autosomal recessive *HCN2*‐related neurodevelopmental disorder^39^; the case exemplified the need for careful variant‐guided inspection of RNA‐seq data, in parallel to pipeline‐based analyses. A total of 3 out of 36 pre‐identified variants (8.3%) were flagged as unanalyzable because the affected genes were not expressed in fibroblasts (see Supplementary Table S1).

### New Diagnostic Findings Enabled by RNA‐Seq

**FIGURE 5:**
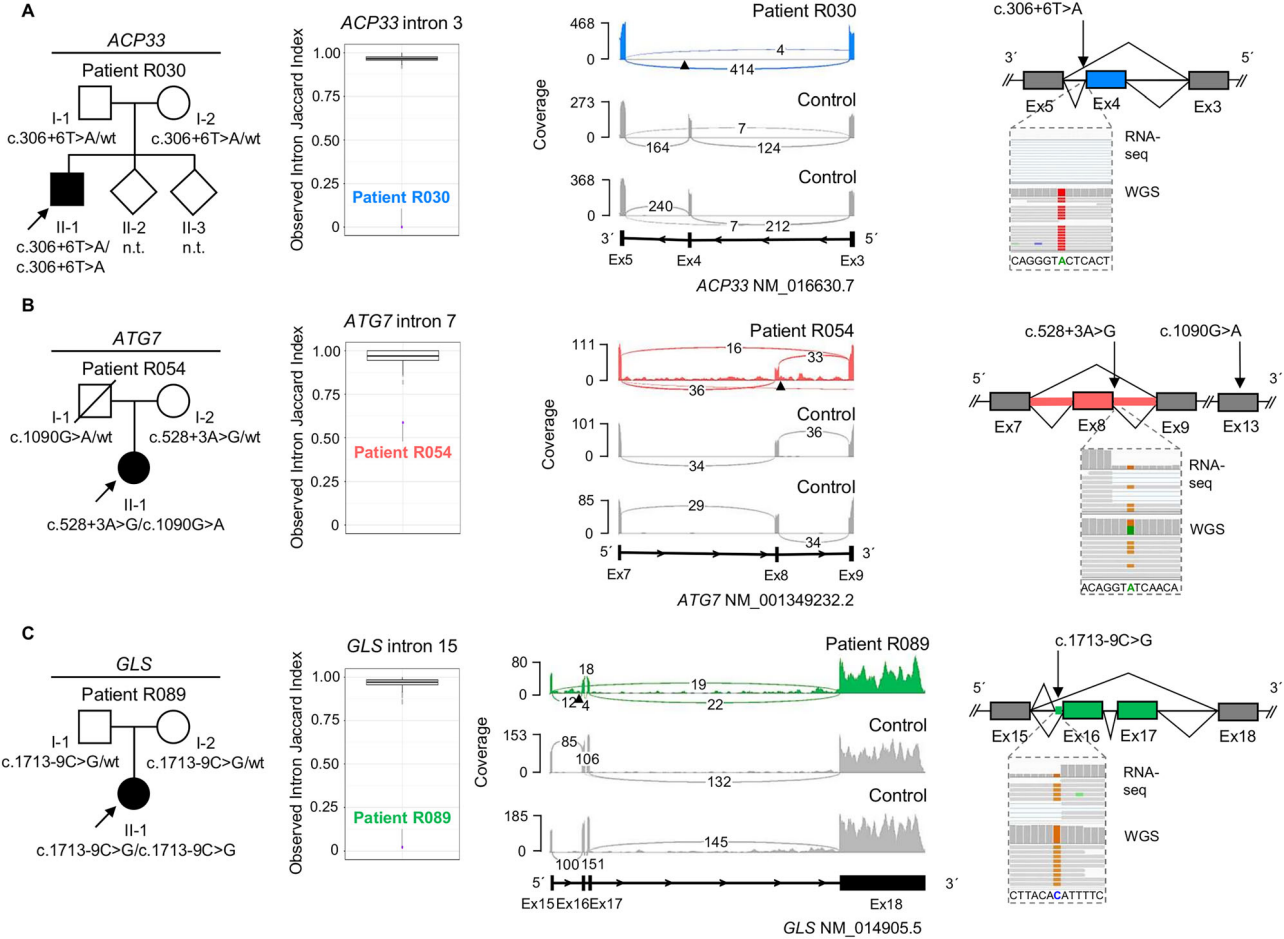
AS event‐directed identification of causative extended splice‐region variants. Pedigrees of patients R030 (A), R054 (B), and R089 (C) with suspected monogenic dystonic syndromes in whom WES/WGS alone did not provide a diagnosis.^2^, ^3^ RNA‐seq data‐based significant splicing‐outlier detection by FRASER 2.0^26^ guided the discovery of homozygous or compound heterozygous extended splice‐region variants that were missed in the initial genomic analyses. Splice metric rank box plots^26^ highlight AS events for *ACP33* (*SPG21*, R030, *blue*), *ATG7* (R054, *red*), and *GLS* (R089, *green*). In the RNA‐seq sashimi plots showing data from the patients and representative control fibroblasts (*gray*), the complete skipping of *ACP33* (*SPG21*) exon 4 as a consequence of the homozygous c.306 + 6T>A variant is in blue, the skipping of *ATG7* exon 8 as well as the retention of introns 7 and 8 as consequences of the heterozygous c.528 + 3A>G variant (found in trans with c.1090G>A) are in red, and the extension of *GLS* exon 16 as well as the skipping of exons 16 plus 17 as consequences of the homozygous c.1713‐9C>G variant are in green. Variant locations are indicated by triangles. The mis‐splicing events are also shown in the schematic representations, along with IGV^19^ screenshots of visualized WGS and RNA‐seq data depicting the read pileups at the mutant chromosomal positions. AS = aberrant splicing; Ex = exon; FRASER = Find RAre Splicing Events in RNA‐seq; IGV = Integrative Genomics Viewer; n.t. = not tested; RNA‐seq = RNA sequencing; WES = whole‐exome sequencing; WGS = whole‐genome sequencing; wt = wild type. [Color figure can be viewed at www.annalsofneurology.org]

**FIGURE 6:**
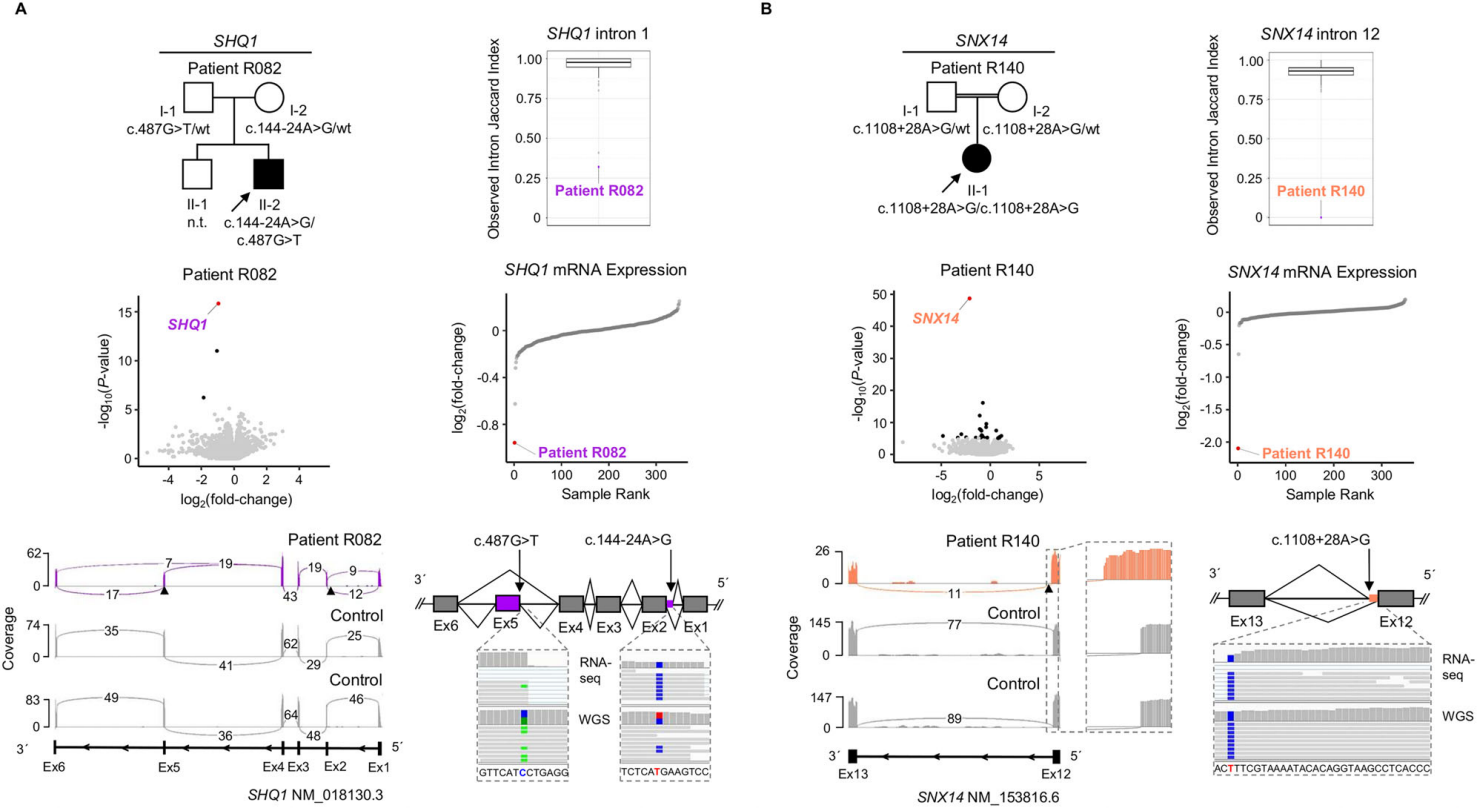
Examples of deep(er) intronic variant‐related diagnoses enabled by automated RNA expression and splicing outlier analyses. Pedigrees of patients R082 (A) and R140 (B) with suspected monogenic dystonic syndromes in whom WES/WGS alone did not provide a diagnosis.^2^, ^3^ RNA‐seq data‐based significant expression‐ and splicing‐outlier detection by OUTRIDER^25^ and FRASER 2.0^26^ guided the discovery of compound heterozygous or homozygous deep(er) intronic variants that were not prioritized in the initial genomic analyses. Splice metric rank box plots^26^ highlight AS events, and RNA expression volcano and sample rank plots highlight AE events for *SHQ1* (R082, *purple*) and *SNX14* (R140, *orange*). In the RNA‐seq sashimi plots showing data from the patients and representative control fibroblasts (*gray*), the extension of *SHQ1* exon 2 as a consequence of the heterozygous c.144‐24A>G variant (found in trans with c.487G>T causing exon‐5 skipping) is in purple, and the extension of *SNX14* exon 12 as a consequence of the homozygous c.1108 + 28A>G variant is in orange. A close‐up of the *SNX14* exon‐12 extension is provided. The mis‐splicing events are also shown in the schematic representations, along with IGV^19^ screenshots of visualized WGS and RNA‐seq data depicting the read pileups at the mutant chromosomal positions. AE = aberrant expression; AS = aberrant splicing; Ex = exon; FRASER = Find RAre Splicing Events in RNA‐seq; IGV = Integrative Genomics Viewer; n.t. = not tested; OUTRIDER = Outlier in RNA‐Seq Finder; RNA‐seq = RNA sequencing; WES = whole‐exome sequencing; WGS = whole‐genome sequencing; wt = wild type. [Color figure can be viewed at www.annalsofneurology.org]

**FIGURE 7:**
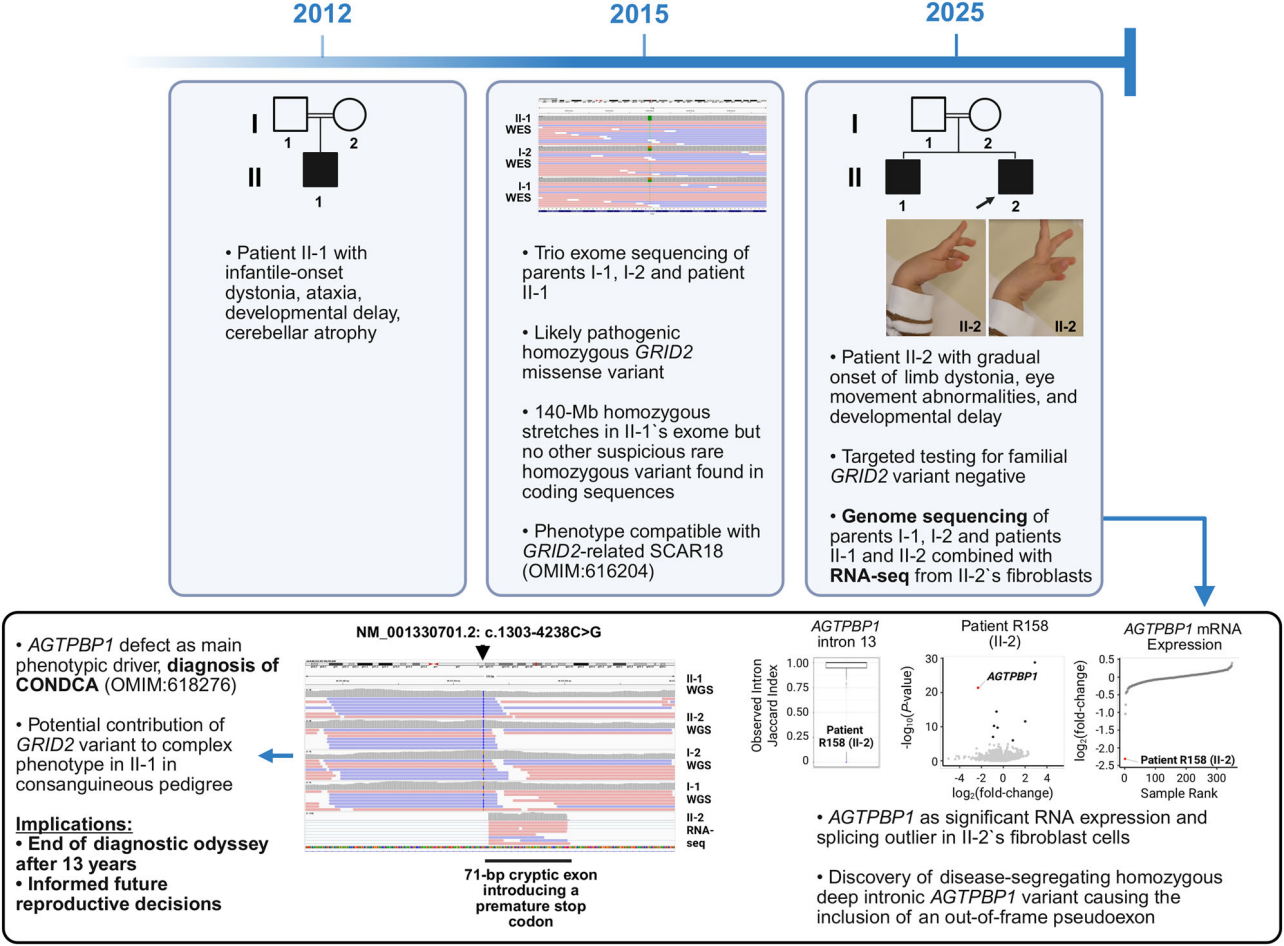
Example of the clinical value of RNA‐seq: bringing an end to the diagnostic odyssey after 13 years. A timeline of the clinical course and applied molecular diagnostics in 2 similarly affected siblings, including patient R158 in this study (II‐2 in the family pedigree, *arrow*; see II‐2's limb dystonia in the clinical photographs). An originally WES‐prioritized homozygous *GRID2* missense variant considered explanatory for the phenotype of the older brother was not identified in R158, ruling out its role as the true (sole) disease‐causing variant in the family. RNA‐seq and WGS reanalysis were instrumental in uncovering the shared monogenic etiology in the siblings: significant AE and AS events^13^ were found for *AGTPBP1* in R158's (II‐2) fibroblasts, resulting from the activation of a pseudoexon through a homozygous deep intronic c.1303‐4238C>G variant (highlighted by a *triangle* in the IGV^19^ screenshot, bottom box). Corresponding splice metric rank box plot^26^ as well as RNA expression volcano and sample rank plots are shown, illustrating the *AGTPBP1* outlier events. The results influenced reproductive decision making after a 13‐year diagnostic journey. AE = aberrant expression; AS = aberrant splicing; CONDCA = neurodegeneration, childhood‐onset, with cerebellar atrophy; IGV = Integrative Genomics Viewer; RNA‐seq = RNA sequencing; WES = whole‐exome sequencing; WGS = whole‐genome sequencing. [Color figure can be viewed at www.annalsofneurology.org]

RNA‐seq aided in establishing new diagnoses in 9 index patients from the “variant‐negative” group, providing a diagnostic uplift of 6.9% (9/131). Causative variants that had not been prioritized in genomic‐data analysis alone were found through significant AS outliers (n = 3) or combined outliers (significant AS plus significant AE, n = 6; see the Table 1). No new diagnoses were made by MAE analysis, and no relevant variants with associated RNA defects were found in non‐dystonia‐related genes, although this was not the particular focus of the present evaluation. These newly solved patients all had more complicated forms of dystonia (8/9 early‐onset, 9/9 non‐focal, and 9/9 combined dystonia); see Supplementary Table S3 for stratification of diagnostic yield according to clinical characteristics. In 3 cases, RNA‐seq directed the prioritization of extended splice‐region variants (±3 bp to ±10 bp region) previously missed by WES/WGS.^2^, ^3^ In patient R030 with dystonia and spastic ataxia, an AS event was found on *ACP33* (*SPG21*), and subsequent RNA‐seq data visualization showed complete skipping of exon 4; WES/WGS‐data reinspection revealed an underlying homozygous c.306 + 6 T>A variant, allowing us to diagnose Mast syndrome (see the Table 1, and Fig 5A). The second case involved R054, a patient with dystonia, ataxia, and intellectual disability; RNA‐seq identified AS of *ATG7*, associated with spinocerebellar ataxia‐31.^20^ On re‐review of R054's WES/WGS data, we identified a heterozygous *ATG7* c.528 + 3A>G variant, associated with exon‐8 skipping, in combination with a c.1090G>A (p.Gly364Ser) missense substitution, which was initially not retained due to lacking additional *ATG7* coding variants. Parental testing demonstrated the bi‐allelic status of the variants, and additional phenotyping revealed cerebellar hypoplasia and characteristic gingival hyperplasia,^20^ confirming the diagnosis (see the Table 1 and Fig 5B). A third case, patient R089, presented with dystonia, developmental delay, and epilepsy and was found to harbor an AS event in *GLS*; with reanalysis‐based detection of a corresponding homozygous c.1713‐9C>G variant causing exon‐16 extension as well as skipping of exons 16 and 17, we diagnosed *GLS*‐associated developmental and epileptic encephalopathy‐71 (see the Table 1 and Fig 5C). Our pipeline also helped to discover deep(er) intronic variants (> ±10 bp from exon‐intron boundaries), diagnosing another 6 cases that could not have been solved without RNA‐seq information. Firstly, a heterozygous c.144‐24A>G variant occurring in trans with a c.487G>T (p.Asp163Tyr) missense variant in *SHQ1* was prioritized from trio‐WGS after AS‐ and AE‐outlier analyses in patient R082. Both changes induced splicing defects (c.144‐24A>G: exon‐2 extension; c.487G>T: exon‐5 skipping), resulting in a significant decrease in *SHQ1* expression (FC = 0.51); R082 presented with dystonia, chorea, and intellectual impairment, consistent with the diagnosis of *SHQ1*‐related neurodevelopmental disorder (see the Table 1 and Fig 6A). Second, patient R140, who manifested developmental delay, cerebellar atrophy, and mixed movement abnormalities with stereotypies and dystonia, had AS and AE (FC = 0.23) hits for *SNX14*; WGS‐data re‐evaluation indicated that the identified *SNX14* mis‐splicing and underexpression resulted from a homozygous c.1108 + 28A>G variant, which led to extension of exon 12; available biochemical‐testing results (elevated urine glycosaminoglycans) complemented the diagnosis of spinocerebellar ataxia‐20 (see the Table 1 and Fig 6B). Third, we clarified the disease cause in patient R158 from a consanguineous family with a similarly affected sibling with dystonia, cerebellar signs, and developmental delay; in the sibling, WES had previously prioritized a homozygous missense variant in *GRID2* (associated with spinocerebellar ataxia‐18), regarded as likely causative for the observed phenotype.^2^ Because the *GRID2* variant was not found in homozygosity upon follow‐up targeted testing in patient R158, a different genetic etiology was considered: through RNA‐seq we found AE of *AGTPBP1* (FC = 0.20), in combination with an AS hit in the same gene; reprioritization of quartet‐WGS variants uncovered a homozygous deep intronic *AGTPBP1* c.1303‐4238C>G alteration shared by both siblings, which induced the inclusion of a cryptic exon; *AGTPBP1*‐related childhood‐onset neurodegeneration with cerebellar atrophy was thus diagnosed (see the Table 1 and Fig 7). Our RNA‐seq analysis pipeline further contributed, in 3 additional index patients, to the characterization of causative intronic variants with disruptive effects on expression and splicing in *ATM*, *SPG11*, and *UFC1*, which have been described in detail in a multi‐omics companion manuscript^3^; for a summary, see the Table 1.

### Incorporation of AbExp Scores

We applied the machine‐learning model AbExp,^27^ which predicts aberrant underexpression in non‐accessible tissues, to causative variants that led to significantly reduced RNA abundance of affected genes in fibroblasts. Benchmarks are shown in Supplementary Figures S4 and S5. The approach estimated that most of the variants for which scores could be obtained from AbExp^27^ (22/25, 88.0%) would operate by loss of expression in different brain regions, including those relevant to dystonia, although the obtained *z*‐scores did not reach the low−/high‐confidence underexpression threshold in 62.6% of all predictions (see Supplementary Table S5 and Supplementary Fig S4). Integrating our present fibroblast RNA‐seq AE results improved the variant‐level predictions, such that combined analysis predicted relevant underexpression in different brain regions for all variants (71.5% high‐confidence underexpression outliers and 28.5% low‐confidence underexpression outliers; see Supplementary Table S5 and Supplementary Fig S4).

## Discussion

Our study provides a systematic evaluation of the diagnostic potential of fibroblast RNA‐seq for a heterogeneous group of dystonias, using a streamlined analysis pipeline. Establishing multiomic pipelines is essential, as standard WES/WGS approaches often fail to provide a diagnosis for many individuals affected by dystonia.^2^, ^3^

Our present framework indicates that (1) fibroblasts capture a broader set of dystonia‐related genes than blood and may therefore be preferable for transcriptomic assessment when tissue choice is possible and (2) advanced bioinformatic tools^13^, ^25^, ^26^ yield a reviewable set of candidate outliers in this approach. Whereas the present exploration focused on a unique set of patients with (ultra)rare dystonic syndromes and diagnostically intractable conditions, recruited to add comprehensive molecular‐characterization analyses in the context of suspected monogenic causation, RNA‐seq may be extendable to the wider dystonia population for unraveling pathogenic variant consequences and genetic etiologies—especially difficult‐to‐interpret non‐coding alterations. Nevertheless, we stress that RNA‐seq complements but does not replace protein‐level or functional assays.

Motivated by applications in other rare‐disease cohorts,^8^, ^9^, ^10^, ^11^, ^12^ we evaluated the detection of a dystonia‐associated gene set in fibroblast RNA‐seq, finding a substantial proportion of genes linked to dystonia (>80% of OMIM dystonia genes^20^). Beyond the utility of fibroblast material for discovering mutational downstream effects in a wide spectrum of genetic subtypes, it promotes evaluation of genes organized into disease pathways.^24^ The observation of the relatively low expression of dopamine‐metabolism–related genes in fibroblasts implies that diagnosticians may need to prioritize conventional biochemical testing over fibroblast RNA‐seq for patients with suspected defects in dopaminergic pathways and unrevealing genomic testing. Although skin biopsies may be a strong candidate source for RNA‐seq‐based diagnostics in dystonia, it should be emphasized that their acquisition is more invasive and can pose burdens to patients. Strategies to streamline fibroblast‐culture establishment for those dystonia‐affected individuals who are most likely to benefit from RNA‐seq analysis (eg, cases with early‐onset, non‐focal combined dystonia, and unrevealing standard genomic testing) should be developed at specialized centers. We point out that the fact that the coverage of dystonia‐related genes was higher in fibroblasts than in blood cannot directly translate into conclusions about improved diagnostic outcomes; future studies are necessary to define the diagnostic impact of this coverage difference.

Integrating the widely used DROP workflow,^13^ each investigated patient sample had a maximum of 3 significant outlier events in OMIM dystonia genes.^20^ This low number of hits made manual review effective. We assessed the capacity of our RNA‐seq analysis pipeline to resolve RNA defects associated with pre‐identified variants. We replicated previous observations^7^, ^10^ that many LoF variants (bi‐ and mono‐allelic, 10/15, 66.7%) caused significant AE detectable by OUTRIDER, suggesting that RNA‐seq and outlier analysis can successfully be used for uncovering dystonia‐relevant underexpression and pathogenic mechanisms, such as haploinsufficiency (eg, for *VPS16*‐related dystonia, 3/3 cases; see Fig 4A, B, and Supplementary Table S1). Whereas our stringent significance cutoff did not retain AE events for 4 cases with heterozygous LoF variants, additional sample rank‐based evaluation^3^, ^32^ constituted a useful complementary strategy. Several other mutation types causally involved in dystonic syndromes, including missense variants, CNVs, repeat expansions, and synonymous changes, variably impacted measurable transcriptional outcomes (aberrant events in 6/21 samples, 28.6%; see Fig 4A and Supplementary Table S1). Thus, complementary fibroblast RNA‐seq serves as a useful strategy to validate the effects of LoF variants across the transcriptome (over two thirds confirmed in this study), whereas the method is less consistently effective for evaluation of other variant types. In this study, there was often no evidence supporting variant pathogenicity for missense changes (88.9%). The RNA‐seq approach can deprioritize suspected transcript‐altering VUS if no splicing and expression defects are detected^7^; however, we find challenges with ruling out the clinical significance of the here‐studied variants without identified RNA aberrations (eg, *SLC30A9* missense VUS; see Supplementary Table S1) because these variants could act via alternative pathogenic mechanisms, such as specific protein‐level perturbation. To formally estimate our framework's discovery potential, we calculated its theoretical diagnostic accuracy (0.68; for details, see Supplementary Table S4), justifying larger follow‐up studies to refine this probabilistic information and the associated impact on patient outcomes.

To prioritize variants evading prior identification by WES/WGS alone, we tested samples with hard‐to‐resolve dystonic conditions. This outlier‐aided approach^10^, ^18^ with subsequent genomic reanalysis facilitated additional diagnoses in 6.9% of cases (9/131). Because we only achieved a modest increase in diagnostic yield, we highlight that future optimizations of RNA‐seq data evaluations and reanalyzes, including integration of additional genomic profiling strategies such as long‐read sequencing, may be necessary. Additionally, other omics layers such as proteomics will increase the diagnostic yield in the future. The currently identified yield indicates that clinical phenotyping and careful case selection for fibroblast‐based RNA diagnostics remains important. We observed the highest effectiveness in reprioritizing previously “missed” causative variants in individuals with early onset, non‐focal combined dystonia and autosomal recessive inheritance. Although the sample size was too small to draw firm conclusions (see Supplementary Table S3), the utility of the present approach may be limited for later‐onset isolated dystonias. We therefore advocate to implement complementary RNA‐seq to aid in interpreting unrevealing genomic data of patients with dystonias of strongly suspected monogenic origin.

New RNA‐seq‐based diagnoses had clinical implications. In all cases, the findings ended diagnostic odysseys and resolved a strong degree of uncertainty that existed across the families. Additionally, in patient R158's family (Fig 7), the discovery of the deep‐intronic homozygous *AGTPBP1* variant influenced reproductive decisions including the option to perform prenatal testing, and, for patient R105, a trial with levodopa was recommended based on the identified *SPG11* variants.^40^

Last, the machine‐learning tool AbExp^27^ predicted that the variants associated with AE in skin would decrease the expression of affected genes in different dystonia‐relevant brain regions. Although AbExp‐based brain expression predictions offer unique insights, many variants currently yield subthreshold effects. Future iterative refinements that jointly leverage expanding RNA‐seq expression datasets across tissues for validation, and improved variant effect predictions on expression are likely to further increase clinical utility.

Limitations of this study included a relatively small cohort size and a restricted set of dystonia‐associated variants and VUSs available for benchmarking analysis. Although our pipeline covered many genes, not all relevant genes could be tested due to non‐expression in fibroblasts (approximately 20%).

Our data encourages the adaptation of RNA‐seq for deepening our understanding of mutational mechanisms, especially expression and splicing defects caused by LoF coding and non‐coding alleles, and improving molecular diagnostics for cases with suspected monogenic dystonias.

## Author Contributions

A.S., I.D., R.J., J.W., E.G., J.G., M.S., and M.Z. contributed to the conception and design of the study. A.S., I.D., T.B., V.A.Y., F.H., E.I., B.A., S.B., D.B., S.B., F.B., M.B., K.D., F.D., P.H., D.H., P.H., A.‐K.J., M.K., R.K., A.K., M.K., L.K., K.K., O.M., M.M.‐B., C.M., A.O., T.R., A.S., U.S., A.M.S., K.V., M.W., H.P., S.B., J.N., R.J., J.W., E.G., J.G., M.S., and M.Z. contributed to the acquisition and/or analysis of the data. A.S., I.D., and M.Z. contributed to drafting the text and/or preparing the figures.

## Potential Conflicts of Interest

V.A.Y., F.B., and C.M. are founders, shareholders and managing directors of OmicsDiscoveries GmbH. The remaining authors have nothing to report.

## Supporting information


**Supplementary** **Data S1.** Supporting Information.

## Data Availability

All genomic sequencing and RNA‐seq data are stored in a repository at the Institute of Human Genetics of TUM University Hospital. Anonymized data is available upon reasonable request from the corresponding author Dr Michael Zech (email: michael.zech@mri.tum.de) according to local ethics review board guidelines.
