# Supplementary material for: Fibroblast Transcriptomics in Molecular Diagnostics of a Comprehensive Dystonia Cohort
Source: Ann Neurol. 2026 Feb 2;99(6):1363–78. doi: 10.1002/ana.78171 (PMC13206262; doi:10.1002/ana.78171)
Supplement: Supplementary file 1 — Supplementary Data S1. Supporting Information. [file ANA-99-1363-s001.pdf]

Supplementary Online Data

Fibroblast transcriptomics in molecular diagnostics of a comprehensive dystonia cohort

Table of contents

Supplementary Online Tables

Supplementary Online Table 1	Summary of pre-identified variants investigated by RNA-seq in this study
Supplementary Online Table 2	Summary of complementary tests
Supplementary Online Table 3	Stratification of RNA-seq-based diagnostic yield according to clinical characteristics
Supplementary Online Table 4	Assessment of the RNA-seq framework`s theoretical discovery potential
Supplementary Online Table 5	Summary of AbExp scores for dystonia-associated variants causing underexpression in skin fibroblasts

Supplementary Online Figures

Supplementary Figure 1	Validation of fibroblast RNA-seq results via proteomics
Supplementary Figure 2	Validation of fibroblast RNA-seq results via Oxford Nanopore Technologies (ONT)-based long read RNA analysis
Supplementary Figure 3	Validation of fibroblast RNA-seq results via qPCR
Supplementary Figure 4	Predicted variant effects on RNA expression in the brain
Supplementary Figure 5	Benchmarking of AbExp performance

Supplementary Online Table

Supplementary Online Table 1 Summary of pre-identified variants[1] investigated by RNA-seq in this study

Index patient/ age/ sex/ genomic sequencing	Phenotype	Gene/ associated disorder (OMIM)	Gene expressed in fibroblasts	Variant(s) (zygosity, clinical significance category: LP/P versus VUS)	RNA phenotype	Tool(s) that identified significant RNA defect	RNA phenotype supporting clinical evaluation of the variant(s)
**Bi-allelic LoF variants**							
R028/ 28y/ F/ WES+WGS	dystonia, cognitive decline	*POLR3A* leukodystrophy, hypomyelinating, 7, with or without oligodontia and/or hypogonadotropic hypogonadism (607694)	yes	NM_007055.4: c.1771-6C>G, p.? (hom, LP/P)	*POLR3A* significant underexpression (FC: 0.57)[3], exon-14 skipping	OUTRIDER	yes: support of LoF effect, support of splice defect
R023/ 10y/ F/ WES	dystonia, DD	*ZNF142* neurodevelopmental disorder with impaired speech and hyperkinetic movements (618425)	yes	NM_001105537.4: c.3175C>T, p.Arg1059* (hom, LP/P)	*ZNF142* significant underexpression (FC: 0.67)	OUTRIDER	yes: support of LoF effect
**Mono-allelic LoF variants**							
R029/ 16y/ M/ WES	dystonia, chorea	*ADCY5* dyskinesia with orofacial involvement, autosomal dominant (606703)	no	NM_183357.2: c.2088+1G>A, p.? (het, LP/P)	unanalyzable (gene not expressed in fibroblasts)	unanalyzable (gene not expressed in fibroblasts)	unanalyzable (gene not expressed in fibroblasts)
R020/ 26y/ M/ WES	dystonia, psychiatric features	*ANK2* *ANK2*-related neurodevelopmental disorder (N/A)	yes	NM_001148.6: c.3804dup, p.Thr1269Hisfs*19 (het, LP/P)	N/A[2]	N/A	(no)[2]
R161/ 43y/ M/ WES+WGS	dystonia, myoclonus, spasticity, DD	*ATP5F1A* mitochondrial complex V (ATP synthase) deficiency, nuclear type 4A (620358)	yes	NM_004046.6: c.1404del, p.Glu469Serfs*3 (het, LP/P)	*ATP5F1A* significant underexpression (FC: 0.63)[3]	OUTRIDER, MAE	yes: support of LoF effect/ haploinsufficiency mechanism

R016/ 14y/ F/ WES+WGS	dystonia, DD	*ATP5F1B* *ATP5F1B*-related dystonia (N/A)	yes	NM_001686.4: c.1074+1G>T, p.? (het, LP/P)	*ATP5F1B* significant underexpression (FC: 0.69)[3], exon-7 skipping, skipping of exons 6 and 7	OUTRIDER	yes: support of LoF effect/ haploinsufficiency mechanism, support of splice defect
R050/ 50y/ F/ WES	dystonia	*CHD3* Snijders Blok-Campeau syndrome (618205)	yes	NM_001005273.3: c.793+1G>A, p.? (het, LP/P)	*CHD3* significant underexpression (FC: 0.65), exon-5 skipping	OUTRIDER, FRASER 2.0	yes: support of LoF effect/ haploinsufficiency mechanism, support of splice defect
R051/ 67y/ M/ WES	dystonia	*EIF4A2* neurodevelopmental disorder with hypotonia and speech delay, with or without seizures (620455)	yes	NM_001967.4: c.896_897del, p.Thr299Serfs*7 (het, LP/P)	*EIF4A2* significant underexpression (FC: 0.74)	OUTRIDER, MAE	yes: support of LoF effect/ haploinsufficiency mechanism
R095/ 11y/ M/ WES+WGS	dystonia, ID, epilepsy	*IRF2BPL* neurodevelopmental disorder with regression, abnormal movements, loss of speech, and seizures (618088)	yes	NM_024496.4: c.189_211dup, p.Gly71Alafs*89 (het, LP/P)	N/A[4]	N/A	no
R154/ 11y/ F/ WES	dystonia, DD, microcephaly	*KMT2B* dystonia 28, childhood-onset (617284)	yes	NM_014727.2: c.610C>T, p.Gln204* (het, LP/P)	N/A[5]	N/A	no
R146/ 7y/ F/ WES+WGS	dystonia, ataxia, spasticity, ID, epilepsy	*MECP2* Rett syndrome (312750)	yes	NM_004992.4: c.1146_1193delinsC, p.Leu383Profs*6 (het, LP/P)	N/A[6]	N/A	no
R083/ 8y/ F/ WES+WGS	dystonia, spasticity	*PTPN1* *PTPN1*-associated interferonopathy (N/A)	yes	NM_002827.4: c.505C>T, p.Arg169* (het, LP/P)	*PTPN1* significant underexpression (FC: 0.65)	OUTRIDER, MAE	yes: support of LoF effect/ haploinsufficiency mechanism
R044/ 26y/ M/ WES	dystonia, psychiatric features	*VPS16* dystonia 30 (619291)	yes	NM_022575.4: c.559C>T, p.Arg187* (het, LP/P)	*VPS16* significant underexpression (FC: 0.58)	OUTRIDER, MAE	yes: support of LoF effect/ haploinsufficiency mechanism
R104/ 41y/ M/ WES	dystonia	*VPS16* dystonia 30 (619291)	yes	NM_022575.4: c.1988_1989insG, p.Asn663Lysfs*2 (het, LP/P)	*VPS16* significant underexpression (FC: 0.59)	OUTRIDER, MAE	yes: support of LoF effect/ haploinsufficiency mechanism
R137/ 30y/ M/ WES	dystonia, psychiatric features	*VPS16* dystonia 30 (619291)	yes	NM_022575.4: c.559C>T, p.Arg187* (het, LP/P)	*VPS16* significant underexpression (FC: 0.59)	OUTRIDER	yes: support of LoF effect/ haploinsufficiency mechanism

Bi-allelic missense variants

R021/ 25y/ F/ WES	dystonia, ataxia, DD, microcephaly	*NUP54* dystonia 37, early-onset, with striatal lesions (620427)	yes	NM_017426.4: c.1073T>G, p.Ile358Ser (hom, LP/P)	N/A	N/A	no
R114/ 15y/ M/ WES	dystonia, myoclonus, ataxia, renal abnormality	*SLC30A9* Birk-Landau-Perez syndrome (617595)	yes	NM_006345.4: c.896C>T, p.Pro299Leu + c.1484A>G, p.Asp495Gly (comp het, VUS + VUS)	N/A	N/A	no

Mono-allelic/hemizygous missense variants

R031/ 45y/ F/ WES+WGS	dystonia, ataxia	*ATP2B2* *ATP2B2*-related neurodevelopmental disorder (N/A)	no	NM_001001331.4: c.3028G>A, p.Glu1010Lys (het, LP/P)	unanalyzable (gene not expressed in fibroblasts)	unanalyzable (gene not expressed in fibroblasts)	unanalyzable (gene not expressed in fibroblasts)
R141/ 16y/ M/ WES	dystonia	*ATP5MC3* dystonia, early-onset, and/or spastic paraplegia (619681)	yes	NM_001689.5: c.318C>G, p.Asn106Lys (het, LP/P)	N/A	N/A	no
R132/ 33y/ M/ WES+WGS	dystonia, myoclonus, DD, ID, epilepsy	*DNM1L* encephalopathy, lethal, due to defective mitochondrial peroxisomal fission 1 (614388)	yes	NM_012062.5: c.176C>T, p.Thr59Ile (het, LP/P)	N/A	N/A	no
R052/ 2y/ M/ WES	dystonia, ID, epilepsy, microcephaly	*MATR3* *MATR3*-related early-onset neurodegeneration (N/A)	yes	NM_018834.6: c.1306G>A, p.Glu436Lys (het, LP/P)	N/A	N/A	no
R072/ 16y/ M/ WES+WGS	dystonia, DD, epilepsy	*MBTPS2* IFAP syndrome with or without BRESHECK syndrome (308205)	yes	NM_015884.4: c.970G>A, p.Ala324Thr (hem, VUS)	*MBTPS2* significant underexpression (FC: 0.65), skipping of exons 6 and 7, partial retention of intron 7	OUTRIDER, FRASER 2.0	yes: demonstration of LoF effect, demonstration of splice defect, RNA evidence supported reclassification (VUS > LP/P)
R130/ 10y/ F/ WES+WGS	dystonia, myoclonus	*SGCE* dystonia-11, myoclonic (159900)	yes	NM_003919.3: c.742T>A, p.Cys248Ser (het, LP/P)	N/A	N/A	no

R085/ 21y/ F/ WES+WGS	dystonia, ataxia, myoclonus, DD, ID	*SLC16A2* Allan-Herndon-Dudley syndrome (300523)	yes	NM_006517.5: c.1025T>C, p.Leu342Pro (het, LP/P)	N/A	N/A	no
**CNVs**							
R139/ 21y/ F/ WES+WGS	dystonia, myoclonus, DD	*NUS1* intellectual developmental disorder, autosomal dominant 55, with seizures (617831)	yes	chr6:117571001-122124500, del chr6q22.1-q22.31, multiple genes including *NUS1* (het, LP/P)	significant underexpression of *NUS1* (FC: 0.51) and 5 other genes in chromosomal region chr6q22.1-q22.31	OUTRIDER	yes: support for the presence of deletion in region chr6q22.1-q22.31, support of LoF effect/ haploinsufficiency mechanism (*NUS1*)
R135/ 47y/ M/ WES+WGS	dystonia, ataxia, spasticity	*SPG7* spastic paraplegia 7, autosomal recessive (607259)	yes	chr16:89596808-89597521, del exon 7 (NM_003119.4) (hom, LP/P)	*SPG7 e*xon-7 skipping	FRASER 2.0	yes: support for the presence of single-exon deletion
**Repeat expansions**							
R057/ 23y/ M/ WES+WGS	dystonia, ataxia	*ATXN8OS* (*SCA8*) spinocerebellar ataxia 8 (608768)	no	chr13:70713515[124] (NR_185841.1), CI: 95-174 CTG units (het, pathological range)	unanalyzable (gene not expressed in fibroblasts)	unanalyzable (gene not expressed in fibroblasts)	unanalyzable (gene not expressed in fibroblasts)
R010/ 19y/ F/ WES+WGS	dystonia, ataxia, myoclonus, epilepsy	*CSTB* epilepsy, progressive myoclonic 1A, Unverricht and Lundborg (254800)	yes	chr21:45196349[38] (NM_000100.4), CI: 27-51 CCCCGCCCCGCG units (allele 1) and 22-42 CCCCGCCCCGCG units (allele 2) (hom, pathological range)	*CSTB* significant underexpression (FC: 0.31)[3]	OUTRIDER	yes: support for presence of repeat expansion, support of LoF effect
R100/ 38y/ M/ WES+WGS	dystonia	*DIP2B* *DIP2B*-related movement disorder (N/A)	yes	chr12:50898784[80] (NM_173602.3), CI: 68-137 GGC units (het, pathological range)	N/A	N/A	no
R152/ 35y/ F/ WES+WGS	dystonia	*FMR1* fragile-X-associated tremor/ataxia syndrome (300623)	yes	chrX:146993568[92] (NM_002024.6), CI: 79-159 CGG units (het, pathological range)	N/A	N/A	no

R034/ 14y/ M/ WES+WGS	dystonia, ataxia, DD, ID	*GLS* global developmental delay, progressive ataxia, and elevated glutamine (618412)	yes	chr2:191745598[138] (NM_014905.5), CI: 115-195 GCA units + c.1197+2T>C, p.? (comp het, pathological range + LP/P)	*GLS* significant underexpression (FC: 0.59), exon-10 extension (c.1197+2T>C)	OUTRIDER, FRASER 2.0	yes: support for presence of repeat expansion, support of splice defect (c.1197+2T>C), support of LoF effect
R151/ 58y/ M/ WES+WGS	dystonia, chorea	*HTT* Huntington disease (143100)	yes	chr4:3076603[40] (NM_001388492.1), 40 CAG units (het, pathological range)	N/A	N/A	no
R101/ 63y/ M/ WES+WGS	dystonia, chorea, cognitive decline, muscle wasting	*PABPN1* oculopharyngeal muscular dystrophy-1 (164300)	yes	chr14:23790681[7] (NM_004643.4), 4 GCG and 3 GCA units (het, pathological range)	N/A	N/A	no
**Synonymous variants (in *trans* with other variant types)**							
R047/ 8y/ M/ WES+WGS	dystonia, epilepsy, DD	*HCN2* *HCN2*-related neurodevelopmental disorder, autosomal recessive (N/A)	yes	NM_001194.4: c.1560C>T, p.Gly520= + del exon 6 (comp het, VUS + LP/P)	IGV-based manual inspection: cryptic splice donor created by c.1560C>T with exon-5 truncation (only visible in 3 reads due to NMD), lowest *HCN2* expression in sample-rank analysis	N/A	yes: support of LoF effect (sample-rank analysis), demonstration of splice defect (IGV), RNA evidence supported reclassification of the synonymous variant (VUS > LP/P)
R149/ 1y/ M/ WES+WGS	dystonia, DD, metabolic decompensation	*SARS2* hyperuricemia, pulmonary hypertension, renal failure, and alkalosis (613845)	yes	NM_017827.4: c.446T>C, p.Leu149Pro + c.627C>T, p.Gly209= (comp het, VUS + VUS)	N/A	N/A	no
R093/ 23y/ M/ WES+WGS	dystonia, ataxia, ID	*TARS2* combined oxidative phosphorylation deficiency 21 (615918)	yes	NM_025150.5: c.774G>T, p.Ser258= + c.1099C>T, p.His367Tyr (comp het, VUS + VUS)	N/A	N/A	no

[1]27 of 36 cases (75.0%) with pre-identified variants were previously published by us: PMIDs: 39937650, 31036918, 40276935, 37485550, 39986310, 32808683, 33998058, 36333996, 34173818, 34954817, 40590478, 37675773.

[2]Lowest *ANK2* expression compared to all other samples that expressed the gene in sample-rank analysis (sample rank: 1/349, FC: 0.51, FDR>0.05).
[3]FC calculated based on new RNA-seq dataset compared to PMIDs: 40276935 and 39937650.
[4]*IRF2BPL* is a single-exon gene and NMD is not expected.
[5]*KMT2B* not significantly underexpressed (sample rank: 25/349, FC: 0.94, FDR>0.05), index patient with milder (atypical) phenotype.
[6]*MECP2* variant located in the last exon with possible NMD escape (sample rank: 60/349, FC: 0.97, FDR>0.05).

Abbreviations: CI, confidence interval (ExpansionHunter); CNV, copy number variant; comp het, compound heterozygous; DD, developmental delay; del, deletion; F, female; FC, fold change; FDR, false-discovery rate; FRASER, Find RAre Splicing Events in RNA-seq; hem, hemizygous; het, heterozygous; hom, homozygous; ID, intellectual disability; IGV, Integrative Genomics Viewer; LoF, loss-of-function; LP/P, likely pathogenic/pathogenic; MAE, mono-allelic expression; M, male; N/A, not applicable/not available; NMD, nonsense-mediated mRNA decay; OMIM, Online Mendelian Inheritance in Man; OUTRIDER, Outlier in RNA-Seq Finder; RNA-seq, RNA sequencing; VUS, variant of uncertain significance, WES, whole-exome sequencing; WGS, whole-genome sequencing; y, years,

Supplementary Online Table 2 Summary of complementary tests[1]

**Patients with pre-identified variants**	
R028, *POLR3A*	segregation in similarly affected sibling
R020, *ANK2*	segregation in 2 similarly affected relatives
R161, *ATP5F1A*	western blotting (reduced ATP5F1A expression), enzymatic assay (impaired ATP synthase activity) (PMID: 40276935)
R016, *ATP5F1B*	enzymatic assay (impaired ATP synthase activity) (PMID: 40276935)
R051, *EIF4A2*	western blotting (reduced EIF4A2 protein expression) (PMID: 37485550)
R083, *PTPN1*	qPCR (*PTPN1* mRNA expression reduced) (PMID: 39986310)
R044, *VPS16*	autophagosomal/lysosomal abnormalities in fibroblast studies (electron microscopy/immunofluorescence)
R104, *VPS16*	autophagosomal/lysosomal abnormalities in fibroblast studies (electron microscopy/immunofluorescence)
R137, *VPS16*	autophagosomal/lysosomal abnormalities in fibroblast studies (electron microscopy/immunofluorescence)
R010, *CSTB*	segregation in similarly affected sibling, proteomics (CSTB protein expression significantly reduced) (PMID: 39937650)
R034, *GLS*	segregation in 2 similarly affected siblings, enzymatic assay (impaired glutaminase activity) (PMID: 39937650)
**Patients with newly prioritized variants from RNA-seq**	
R030, *ACP33*	proteomics (ACP33 protein expression significantly reduced) (see **Suppl.Fig.1**)
R054, *ATG7*	Oxford Nanopore Technologies (ONT)-based long-read RNA sequencing (see **Suppl.Fig.2**): confirmation of splicing defect
R089, *GLS*	proteomics (GLS protein expression significantly reduced) (see **Suppl.Fig.1**)
R082, *SHQ1*	Oxford Nanopore Technologies (ONT)-based long-read RNA sequencing (see **Suppl.Fig.2**): confirmation of splicing defects
R140, *SNX14*	glucosaminoglycans in urine elevated
R158, *AGTPBP1*	proteomics (AGTPBP1 protein expression significantly reduced) (see **Suppl.Fig.1**), qPCR (*AGTPBP1* mRNA expression reduced) (see **Suppl.Fig.3**)
R134, *ATM*	Proteomics (ATM protein expression significantly reduced) (see **Suppl.Fig.1**) (PMID: 39937650), qPCR (*ATM* mRNA expression reduced) (see **Suppl.Fig.3**), AFP in blood elevated

R105, *SPG11*	proteomics (SPG11 protein expression significantly reduced) (see **Suppl.Fig.1**) (PMID: 39937650)
R058, *UFC1*	proteomics (UFC1 protein expression significantly reduced) (see **Suppl.Fig.1**) (PMID: 39937650)

[1]A comprehensive array of suitable complementary tests was performed to support, validate, and/or confirm the results from RNA-seq in patients with pre-identified variants and patients with newly prioritized causative variants after RNA-seq-guided re-analysis of genomic data.

Supplementary Online Table 3 Stratification of RNA-seq-based diagnostic yield[1] according to clinical characteristics

Predictor	Method[1]	*n* patients	odds ratio (CI)	*p*-value	Category	Diagnostic yield
Coexisting features	firth	131	7.41 (0.90 - 964.41)	0.067	isolated	0/34 (0.0%)
					combined	9/97 (9.3%)
Dystonia distribution	firth	131	0.63 (0.00 - 5.70)	0.74	non-focal	9/122 (7.4%)
					focal	0/9 (0.0%)
Specific comorbidity	firth	131	1.98 (0.54 - 8.65)	0.309	none	3/66 (4.5%)
					ID/Epilepsy	6/65 (9.2%)
Neuroimaging abnormality	firth	117[2]	2.64 (0.71 - 10.39)	0.147	No	4/78 (5.1%)
					Yes	5/39 (12.8%)
Dystonia age at onset	glm	131	0.98 (0.92 - 1.05)	0.605	N/A	N/A

[1]Logistic regression analysis was performed for the “variant-negative“ group (*n* = 131).
[2]Neuroimaging results available for *n* = 117 patients.

Abbreviations: CI, confidence interval; ID, intellectual disability; N/A, not applicable/not available.

Supplementary Online Table 4 Assessment of the RNA-seq framework`s theoretical discovery potential

True positives	24
False negatives	21
False positives	32
True negatives	90
Accuracy	0.6826
Sensitivity	0.5333
Specificity	0.7377
Positive predictive value (PPV)	0.4286
Negative predictive value (NPV)	0.8108

Using the set of known variants as ground truth, we identified 24 true positives and 21 false negatives, corresponding to a sensitivity of 0.5333 (detected ~53% of known variants). Among loci without a known variant, our approach yielded 90 true negatives and 32 false positives, giving a specificity of 0.7377. Overall performance was accuracy = 0.6826, with PPV = 0.4286 and NPV = 0.8108.

Supplementary Online Table 5 Summary of AbExp scores for dystonia-associated variants causing underexpression in skin fibroblasts

Chromosome	Start	End	Ref	Alt	Gene	Tissue	Tissue Type	AbExp (z-score)	RNA AbExp (logistic regression)
chr1	161127085	161127098	TGAGTTTGACATC	T	ENSG00000143222	Brain - Caudate (basal ganglia)	Brain	-0.1525284	0.49196146
chr1	161127085	161127098	TGAGTTTGACATC	T	ENSG00000143222	Brain - Cerebellar Hemisphere	Brain	-0.1525284	0.49196146
chr1	161127085	161127098	TGAGTTTGACATC	T	ENSG00000143222	Brain - Cortex	Brain	-0.1525284	0.49196146
chr1	161127085	161127098	TGAGTTTGACATC	T	ENSG00000143222	Brain - Frontal Cortex (BA9)	Brain	-0.1525284	0.49196146
chr1	161127085	161127098	TGAGTTTGACATC	T	ENSG00000143222	Brain - Hippocampus	Brain	-0.1525284	0.49196146
chr1	161127085	161127098	TGAGTTTGACATC	T	ENSG00000143222	Brain - Hypothalamus	Brain	-0.1525284	0.49196146
chr1	161127085	161127098	TGAGTTTGACATC	T	ENSG00000143222	Brain - Nucleus accumbens (basal ganglia)	Brain	-0.1525284	0.49196146
chr1	161127085	161127098	TGAGTTTGACATC	T	ENSG00000143222	Brain - Putamen (basal ganglia)	Brain	-0.1525284	0.49196146
chr1	161127085	161127098	TGAGTTTGACATC	T	ENSG00000143222	Brain - Spinal cord (cervical c-1)	Brain	-0.1525284	0.49196146
chr1	161127085	161127098	TGAGTTTGACATC	T	ENSG00000143222	Brain - Substantia nigra	Brain	-0.1512097	0.49194345
chr1	161127123	161127124	G	A	ENSG00000143222	Brain - Caudate (basal ganglia)	Brain	-0.1285439	0.49163396
chr1	161127123	161127124	G	A	ENSG00000143222	Brain - Cerebellar Hemisphere	Brain	-0.1396183	0.49178518
chr1	161127123	161127124	G	A	ENSG00000143222	Brain - Cortex	Brain	-0.1285439	0.49163396
chr1	161127123	161127124	G	A	ENSG00000143222	Brain - Frontal Cortex (BA9)	Brain	-0.1285439	0.49163396
chr1	161127123	161127124	G	A	ENSG00000143222	Brain - Hippocampus	Brain	-0.1285439	0.49163396
chr1	161127123	161127124	G	A	ENSG00000143222	Brain - Hypothalamus	Brain	-0.1285439	0.49163396
chr1	161127123	161127124	G	A	ENSG00000143222	Brain - Nucleus accumbens (basal ganglia)	Brain	-0.1285439	0.49163396
chr1	161127123	161127124	G	A	ENSG00000143222	Brain - Putamen (basal ganglia)	Brain	-0.1285439	0.49163396
chr1	161127123	161127124	G	A	ENSG00000143222	Brain - Spinal cord (cervical c-1)	Brain	-0.1285439	0.49163396
chr1	161127123	161127124	G	A	ENSG00000143222	Brain - Substantia nigra	Brain	-0.1272251	0.49161595
chr2	191788710	191788711	T	C	ENSG00000115419	Brain - Caudate (basal ganglia)	Brain	-2.704433	0.24553125
chr2	191788710	191788711	T	C	ENSG00000115419	Brain - Cerebellar Hemisphere	Brain	-2.8238558	0.2574249
chr2	191788710	191788711	T	C	ENSG00000115419	Brain - Cortex	Brain	-3.3163621	0.31028616
chr2	191788710	191788711	T	C	ENSG00000115419	Brain - Frontal Cortex (BA9)	Brain	-2.3989541	0.21682993
chr2	191788710	191788711	T	C	ENSG00000115419	Brain - Hippocampus	Brain	-2.1689678	0.19687586

chr2	191788710	191788711	T	C	ENSG00000115419	Brain - Hypothalamus	Brain	-2.2358091	0.20252838
chr2	191788710	191788711	T	C	ENSG00000115419	Brain - Nucleus accumbens (basal ganglia)	Brain	-2.0701201	0.18873634
chr2	191788710	191788711	T	C	ENSG00000115419	Brain - Putamen (basal ganglia)	Brain	-2.3203888	0.20985326
chr2	191788710	191788711	T	C	ENSG00000115419	Brain - Spinal cord (cervical c-1)	Brain	-1.4335609	0.14245065
chr2	191788710	191788711	T	C	ENSG00000115419	Brain - Substantia nigra	Brain	-1.8080277	0.16841104
chr2	219508063	219508064	G	A	ENSG00000115568	Brain - Caudate (basal ganglia)	Brain	-3.29402	0.49736537
chr2	219508063	219508064	G	A	ENSG00000115568	Brain - Cerebellar Hemisphere	Brain	-2.3077184	0.46547442
chr2	219508063	219508064	G	A	ENSG00000115568	Brain - Cortex	Brain	-2.671205	0.47720839
chr2	219508063	219508064	G	A	ENSG00000115568	Brain - Frontal Cortex (BA9)	Brain	-2.7664803	0.48028874
chr2	219508063	219508064	G	A	ENSG00000115568	Brain - Hippocampus	Brain	-3.3785098	0.50010194
chr2	219508063	219508064	G	A	ENSG00000115568	Brain - Hypothalamus	Brain	-2.7681826	0.48034379
chr2	219508063	219508064	G	A	ENSG00000115568	Brain - Nucleus accumbens (basal ganglia)	Brain	-2.6200048	0.47555374
chr2	219508063	219508064	G	A	ENSG00000115568	Brain - Putamen (basal ganglia)	Brain	-3.1809081	0.49370202
chr2	219508063	219508064	G	A	ENSG00000115568	Brain - Spinal cord (cervical c-1)	Brain	-2.3156533	0.46573021
chr2	219508063	219508064	G	A	ENSG00000115568	Brain - Substantia nigra	Brain	-2.6542191	0.4766594
chr3	72881631	72881632	C	A	ENSG00000144736	Brain - Cerebellar Hemisphere	Brain	-0.1849922	0.87907336
chr3	72881631	72881632	C	A	ENSG00000144736	Brain - Frontal Cortex (BA9)	Brain	-0.2238248	0.87766522
chr3	72881631	72881632	C	A	ENSG00000144736	Brain - Hippocampus	Brain	-0.1863109	0.87902577
chr3	72881631	72881632	C	A	ENSG00000144736	Brain - Hypothalamus	Brain	-0.1863109	0.87902577
chr3	72881631	72881632	C	A	ENSG00000144736	Brain - Spinal cord (cervical c-1)	Brain	-0.1863109	0.87902577
chr3	72881631	72881632	C	A	ENSG00000144736	Brain - Substantia nigra	Brain	-0.1863109	0.87902577
chr3	72893597	72893598	T	C	ENSG00000144736	Brain - Cerebellar Hemisphere	Brain	-0.0633094	0.88339554
chr3	72893597	72893598	T	C	ENSG00000144736	Brain - Frontal Cortex (BA9)	Brain	-0.1282273	0.88110659
chr3	72893597	72893598	T	C	ENSG00000144736	Brain - Hippocampus	Brain	-0.0646281	0.88334942
chr3	72893597	72893598	T	C	ENSG00000144736	Brain - Hypothalamus	Brain	-0.0646281	0.88334942
chr3	72893597	72893598	T	C	ENSG00000144736	Brain - Spinal cord (cervical c-1)	Brain	-0.0646281	0.88334942

chr3	72893597	72893598	T	C	ENSG00000144736	Brain - Substantia nigra	Brain	-0.0646281	0.88334942
chr3	186505036	186505039	TCA	T	ENSG00000156976	Brain - Caudate (basal ganglia)	Brain	-1.0124405	0.07144223
chr3	186505036	186505039	TCA	T	ENSG00000156976	Brain - Cerebellar Hemisphere	Brain	-1.0551993	0.07332787
chr3	186505036	186505039	TCA	T	ENSG00000156976	Brain - Cortex	Brain	-1.0438588	0.07282335
chr3	186505036	186505039	TCA	T	ENSG00000156976	Brain - Frontal Cortex (BA9)	Brain	-1.0335593	0.0723679
chr3	186505036	186505039	TCA	T	ENSG00000156976	Brain - Hippocampus	Brain	-1.2083797	0.08046479
chr3	186505036	186505039	TCA	T	ENSG00000156976	Brain - Hypothalamus	Brain	-1.3874482	0.08960766
chr3	186505036	186505039	TCA	T	ENSG00000156976	Brain - Nucleus accumbens (basal ganglia)	Brain	-1.2651689	0.08326814
chr3	186505036	186505039	TCA	T	ENSG00000156976	Brain - Putamen (basal ganglia)	Brain	-1.0480786	0.07301071
chr3	186505036	186505039	TCA	T	ENSG00000156976	Brain - Spinal cord (cervical c-1)	Brain	-1.0480786	0.07301071
chr3	186505036	186505039	TCA	T	ENSG00000156976	Brain - Substantia nigra	Brain	-1.0480786	0.07301071
chr6	86256801	86256802	T	C	ENSG00000135317	Brain - Caudate (basal ganglia)	Brain	-0.5750492	0.99997611
chr6	86256801	86256802	T	C	ENSG00000135317	Brain - Cerebellar Hemisphere	Brain	-0.4199903	0.99998302
chr6	86256801	86256802	T	C	ENSG00000135317	Brain - Cortex	Brain	-0.5750492	0.99997611
chr6	86256801	86256802	T	C	ENSG00000135317	Brain - Frontal Cortex (BA9)	Brain	-0.4199903	0.99998302
chr6	86256801	86256802	T	C	ENSG00000135317	Brain - Hippocampus	Brain	-0.2730694	0.99998771
chr6	86256801	86256802	T	C	ENSG00000135317	Brain - Hypothalamus	Brain	-0.5750492	0.99997611
chr6	86256801	86256802	T	C	ENSG00000135317	Brain - Nucleus accumbens (basal ganglia)	Brain	-0.5750492	0.99997611
chr6	86256801	86256802	T	C	ENSG00000135317	Brain - Putamen (basal ganglia)	Brain	-0.4199903	0.99998302
chr6	86256801	86256802	T	C	ENSG00000135317	Brain - Spinal cord (cervical c-1)	Brain	-0.5750492	0.99997611
chr6	86256801	86256802	T	C	ENSG00000135317	Brain - Substantia nigra	Brain	-0.5750492	0.99997611
chr9	88252526	88252527	G	C	ENSG00000135049	Brain - Caudate (basal ganglia)	Brain	-0.0679415	0.99577247
chr9	88252526	88252527	G	C	ENSG00000135049	Brain - Cerebellar Hemisphere	Brain	-0.0688679	0.99576858
chr9	88252526	88252527	G	C	ENSG00000135049	Brain - Cortex	Brain	-0.0675492	0.99577412
chr9	88252526	88252527	G	C	ENSG00000135049	Brain - Frontal Cortex (BA9)	Brain	-0.0675492	0.99577412
chr9	88252526	88252527	G	C	ENSG00000135049	Brain - Hippocampus	Brain	-0.0681796	0.99577147

chr9	88252526	88252527	G	C	ENSG00000135049	Brain - Hypothalamus	Brain	-0.0681796	0.99577147
chr9	88252526	88252527	G	C	ENSG00000135049	Brain - Nucleus accumbens (basal ganglia)	Brain	-0.0688679	0.99576858
chr9	88252526	88252527	G	C	ENSG00000135049	Brain - Putamen (basal ganglia)	Brain	-0.0681796	0.99577147
chr9	88252526	88252527	G	C	ENSG00000135049	Brain - Spinal cord (cervical c-1)	Brain	-0.0675492	0.99577412
chr9	88252526	88252527	G	C	ENSG00000135049	Brain - Substantia nigra	Brain	-0.0681796	0.99577147
chr10	79769438	79769439	G	C	ENSG00000148606	Brain - Caudate (basal ganglia)	Brain	-0.0309617	0.93029199
chr10	79769438	79769439	G	C	ENSG00000148606	Brain - Cerebellar Hemisphere	Brain	-0.0246999	0.93047242
chr10	79769438	79769439	G	C	ENSG00000148606	Brain - Cortex	Brain	-0.0246999	0.93047242
chr10	79769438	79769439	G	C	ENSG00000148606	Brain - Frontal Cortex (BA9)	Brain	-0.0246999	0.93047242
chr10	79769438	79769439	G	C	ENSG00000148606	Brain - Hippocampus	Brain	-0.0397982	0.93003663
chr10	79769438	79769439	G	C	ENSG00000148606	Brain - Hypothalamus	Brain	-0.0411169	0.92999845
chr10	79769438	79769439	G	C	ENSG00000148606	Brain - Nucleus accumbens (basal ganglia)	Brain	-0.0411169	0.92999845
chr10	79769438	79769439	G	C	ENSG00000148606	Brain - Putamen (basal ganglia)	Brain	-0.0387328	0.93006747
chr10	79769438	79769439	G	C	ENSG00000148606	Brain - Spinal cord (cervical c-1)	Brain	-0.0399304	0.9300328
chr10	79769438	79769439	G	C	ENSG00000148606	Brain - Substantia nigra	Brain	-0.0397982	0.93003663
chr11	108144273	108144274	G	T	ENSG00000149311	Brain - Caudate (basal ganglia)	Brain	-0.0061888	0.99255135
chr11	108144273	108144274	G	T	ENSG00000149311	Brain - Cerebellar Hemisphere	Brain	-0.0061888	0.99255135
chr11	108144273	108144274	G	T	ENSG00000149311	Brain - Cortex	Brain	-0.0048701	0.9925599
chr11	108144273	108144274	G	T	ENSG00000149311	Brain - Frontal Cortex (BA9)	Brain	-0.0061888	0.99255135
chr11	108144273	108144274	G	T	ENSG00000149311	Brain - Hippocampus	Brain	-0.0055005	0.99255581
chr11	108144273	108144274	G	T	ENSG00000149311	Brain - Hypothalamus	Brain	-0.0061888	0.99255135
chr11	108144273	108144274	G	T	ENSG00000149311	Brain - Nucleus accumbens (basal ganglia)	Brain	-0.0061888	0.99255135
chr11	108144273	108144274	G	T	ENSG00000149311	Brain - Putamen (basal ganglia)	Brain	-0.0048701	0.9925599
chr11	108144273	108144274	G	T	ENSG00000149311	Brain - Spinal cord (cervical c-1)	Brain	-0.0050023	0.99255904
chr11	108144273	108144274	G	T	ENSG00000149311	Brain - Substantia nigra	Brain	-0.0055005	0.99255581
chr11	108144277	108144278	A	C	ENSG00000149311	Brain - Caudate (basal ganglia)	Brain	-0.0061888	0.99255135

chr11	108144277	108144278	A	C	ENSG00000149311	Brain - Cerebellar Hemisphere	Brain	-0.0061888	0.99255135
chr11	108144277	108144278	A	C	ENSG00000149311	Brain - Cortex	Brain	-0.0048701	0.9925599
chr11	108144277	108144278	A	C	ENSG00000149311	Brain - Frontal Cortex (BA9)	Brain	-0.0061888	0.99255135
chr11	108144277	108144278	A	C	ENSG00000149311	Brain - Hippocampus	Brain	-0.0055005	0.99255581
chr11	108144277	108144278	A	C	ENSG00000149311	Brain - Hypothalamus	Brain	-0.0061888	0.99255135
chr11	108144277	108144278	A	C	ENSG00000149311	Brain - Nucleus accumbens (basal ganglia)	Brain	-0.0061888	0.99255135
chr11	108144277	108144278	A	C	ENSG00000149311	Brain - Putamen (basal ganglia)	Brain	-0.0048701	0.9925599
chr11	108144277	108144278	A	C	ENSG00000149311	Brain - Spinal cord (cervical c-1)	Brain	-0.0050023	0.99255904
chr11	108144277	108144278	A	C	ENSG00000149311	Brain - Substantia nigra	Brain	-0.0055005	0.99255581
chr12	57036240	57036241	C	A	ENSG00000110955	Brain - Caudate (basal ganglia)	Brain	-2.0111654	0.98338017
chr12	57036240	57036241	C	A	ENSG00000110955	Brain - Cerebellar Hemisphere	Brain	-1.8934632	0.98548117
chr12	57036240	57036241	C	A	ENSG00000110955	Brain - Cortex	Brain	-1.8066246	0.98686133
chr12	57036240	57036241	C	A	ENSG00000110955	Brain - Frontal Cortex (BA9)	Brain	-1.8934632	0.98548117
chr12	57036240	57036241	C	A	ENSG00000110955	Brain - Hippocampus	Brain	-1.7203169	0.98810459
chr12	57036240	57036241	C	A	ENSG00000110955	Brain - Hypothalamus	Brain	-1.9592438	0.9843415
chr12	57036240	57036241	C	A	ENSG00000110955	Brain - Nucleus accumbens (basal ganglia)	Brain	-2.0111654	0.98338017
chr12	57036240	57036241	C	A	ENSG00000110955	Brain - Putamen (basal ganglia)	Brain	-1.7732249	0.98735697
chr12	57036240	57036241	C	A	ENSG00000110955	Brain - Spinal cord (cervical c-1)	Brain	-1.9488052	0.98452806
chr12	57036240	57036241	C	A	ENSG00000110955	Brain - Substantia nigra	Brain	-1.7796156	0.98726358
chr15	44898316	44898317	T	C	ENSG00000104133	Brain - Caudate (basal ganglia)	Brain	0.04196113	0.99998856
chr15	44898316	44898317	T	C	ENSG00000104133	Brain - Cerebellar Hemisphere	Brain	-0.5064238	0.99996411
chr15	44898316	44898317	T	C	ENSG00000104133	Brain - Cortex	Brain	0.04196113	0.99998856
chr15	44898316	44898317	T	C	ENSG00000104133	Brain - Frontal Cortex (BA9)	Brain	-0.0598465	0.99998585
chr15	44898316	44898317	T	C	ENSG00000104133	Brain - Hippocampus	Brain	0.04196113	0.99998856
chr15	44898316	44898317	T	C	ENSG00000104133	Brain - Hypothalamus	Brain	0.04196113	0.99998856
chr15	44898316	44898317	T	C	ENSG00000104133	Brain - Nucleus accumbens (basal ganglia)	Brain	-0.0598465	0.99998585

chr15	44898316	44898317	T	C	ENSG00000104133	Brain - Putamen (basal ganglia)	Brain	-0.0598465	0.99998585
chr15	44898316	44898317	T	C	ENSG00000104133	Brain - Spinal cord (cervical c-1)	Brain	0.04196113	0.99998856
chr15	44898316	44898317	T	C	ENSG00000104133	Brain - Substantia nigra	Brain	0.04196113	0.99998856
chr15	44943909	44943910	G	C	ENSG00000104133	Brain - Caudate (basal ganglia)	Brain	-0.973936	0.99990488
chr15	44943909	44943910	G	C	ENSG00000104133	Brain - Cerebellar Hemisphere	Brain	-0.973936	0.99990488
chr15	44943909	44943910	G	C	ENSG00000104133	Brain - Cortex	Brain	-0.973936	0.99990488
chr15	44943909	44943910	G	C	ENSG00000104133	Brain - Frontal Cortex (BA9)	Brain	-0.973936	0.99990488
chr15	44943909	44943910	G	C	ENSG00000104133	Brain - Hippocampus	Brain	-0.973936	0.99990488
chr15	44943909	44943910	G	C	ENSG00000104133	Brain - Hypothalamus	Brain	-0.973936	0.99990488
chr15	44943909	44943910	G	C	ENSG00000104133	Brain - Nucleus accumbens (basal ganglia)	Brain	-0.973936	0.99990488
chr15	44943909	44943910	G	C	ENSG00000104133	Brain - Putamen (basal ganglia)	Brain	-0.973936	0.99990488
chr15	44943909	44943910	G	C	ENSG00000104133	Brain - Spinal cord (cervical c-1)	Brain	-0.973936	0.99990488
chr15	44943909	44943910	G	C	ENSG00000104133	Brain - Substantia nigra	Brain	-0.973936	0.99990488
chr17	7796887	7796888	G	A	ENSG00000170004	Brain - Caudate (basal ganglia)	Brain	-0.1271223	0.13864794
chr17	7796887	7796888	G	A	ENSG00000170004	Brain - Cerebellar Hemisphere	Brain	0.27743361	0.12036288
chr17	7796887	7796888	G	A	ENSG00000170004	Brain - Cortex	Brain	-0.9909696	0.18546925
chr17	7796887	7796888	G	A	ENSG00000170004	Brain - Frontal Cortex (BA9)	Brain	-0.8266733	0.17570972
chr17	7796887	7796888	G	A	ENSG00000170004	Brain - Hippocampus	Brain	-0.1066728	0.13767029
chr17	7796887	7796888	G	A	ENSG00000170004	Brain - Hypothalamus	Brain	-0.2453936	0.14441696
chr17	7796887	7796888	G	A	ENSG00000170004	Brain - Nucleus accumbens (basal ganglia)	Brain	-0.014735	0.1333463
chr17	7796887	7796888	G	A	ENSG00000170004	Brain - Putamen (basal ganglia)	Brain	-0.1133886	0.13799072
chr17	7796887	7796888	G	A	ENSG00000170004	Brain - Spinal cord (cervical c-1)	Brain	-0.0767163	0.13624859
chr17	7796887	7796888	G	A	ENSG00000170004	Brain - Substantia nigra	Brain	-0.1227029	0.13843616
chr18	43666102	43666104	CA	C	ENSG00000152234	Brain - Caudate (basal ganglia)	Brain	-3.0394813	0.8838417
chr18	43666102	43666104	CA	C	ENSG00000152234	Brain - Cerebellar Hemisphere	Brain	-3.3972784	0.85157828
chr18	43666102	43666104	CA	C	ENSG00000152234	Brain - Cortex	Brain	-3.308043	0.86025837

chr18	43666102	43666104	CA	C	ENSG00000152234	Brain - Frontal Cortex (BA9)	Brain	-3.0394813	0.8838417
chr18	43666102	43666104	CA	C	ENSG00000152234	Brain - Hippocampus	Brain	-2.660154	0.91121822
chr18	43666102	43666104	CA	C	ENSG00000152234	Brain - Hypothalamus	Brain	-2.6416618	0.91239146
chr18	43666102	43666104	CA	C	ENSG00000152234	Brain - Nucleus accumbens (basal ganglia)	Brain	-3.0394813	0.8838417
chr18	43666102	43666104	CA	C	ENSG00000152234	Brain - Putamen (basal ganglia)	Brain	-2.8406742	0.89900126
chr18	43666102	43666104	CA	C	ENSG00000152234	Brain - Spinal cord (cervical c-1)	Brain	-3.0394813	0.8838417
chr18	43666102	43666104	CA	C	ENSG00000152234	Brain - Substantia nigra	Brain	-2.3418588	0.9295453
chr20	2845684	2845685	A	AG	ENSG00000215305	Brain - Caudate (basal ganglia)	Brain	-2.4216598	0.3079829
chr20	2845684	2845685	A	AG	ENSG00000215305	Brain - Cerebellar Hemisphere	Brain	-2.4216598	0.3079829
chr20	2845684	2845685	A	AG	ENSG00000215305	Brain - Cortex	Brain	-2.4188755	0.30776445
chr20	2845684	2845685	A	AG	ENSG00000215305	Brain - Frontal Cortex (BA9)	Brain	-2.5493245	0.31809091
chr20	2845684	2845685	A	AG	ENSG00000215305	Brain - Hippocampus	Brain	-2.3069544	0.29905499
chr20	2845684	2845685	A	AG	ENSG00000215305	Brain - Hypothalamus	Brain	-2.4216598	0.3079829
chr20	2845684	2845685	A	AG	ENSG00000215305	Brain - Nucleus accumbens (basal ganglia)	Brain	-2.3669026	0.30370249
chr20	2845684	2845685	A	AG	ENSG00000215305	Brain - Putamen (basal ganglia)	Brain	-2.0496773	0.27958247
chr20	2845684	2845685	A	AG	ENSG00000215305	Brain - Spinal cord (cervical c-1)	Brain	-2.3617116	0.30329844
chr20	2845684	2845685	A	AG	ENSG00000215305	Brain - Substantia nigra	Brain	-2.3406011	0.30165844
chr20	2841437	2841438	C	T	ENSG00000215305	Brain - Caudate (basal ganglia)	Brain	-2.5511674	0.36090352
chr20	2841437	2841438	C	T	ENSG00000215305	Brain - Cerebellar Hemisphere	Brain	-2.5600501	0.361516
chr20	2841437	2841438	C	T	ENSG00000215305	Brain - Cortex	Brain	-2.5657891	0.36191195
chr20	2841437	2841438	C	T	ENSG00000215305	Brain - Frontal Cortex (BA9)	Brain	-2.5657891	0.36191195
chr20	2841437	2841438	C	T	ENSG00000215305	Brain - Hippocampus	Brain	-2.5569064	0.36129918
chr20	2841437	2841438	C	T	ENSG00000215305	Brain - Hypothalamus	Brain	-2.5511674	0.36090352
chr20	2841437	2841438	C	T	ENSG00000215305	Brain - Nucleus accumbens (basal ganglia)	Brain	-2.5569064	0.36129918
chr20	2841437	2841438	C	T	ENSG00000215305	Brain - Putamen (basal ganglia)	Brain	-2.0199462	0.32515267
chr20	2841437	2841438	C	T	ENSG00000215305	Brain - Spinal cord (cervical c-1)	Brain	-2.5511674	0.36090352

chr20	2841437	2841438	C	T	ENSG00000215305	Brain - Substantia nigra	Brain	-2.3874208	0.34969553
chr20	2841437	2841438	C	T	ENSG00000215305	Brain - Caudate (basal ganglia)	Brain	-2.5511674	0.34352627
chr20	2841437	2841438	C	T	ENSG00000215305	Brain - Cerebellar Hemisphere	Brain	-2.5600501	0.34418078
chr20	2841437	2841438	C	T	ENSG00000215305	Brain - Cortex	Brain	-2.5657891	0.34460396
chr20	2841437	2841438	C	T	ENSG00000215305	Brain - Frontal Cortex (BA9)	Brain	-2.5657891	0.34460396
chr20	2841437	2841438	C	T	ENSG00000215305	Brain - Hippocampus	Brain	-2.5569064	0.34394907
chr20	2841437	2841438	C	T	ENSG00000215305	Brain - Hypothalamus	Brain	-2.5511674	0.34352627
chr20	2841437	2841438	C	T	ENSG00000215305	Brain - Nucleus accumbens (basal ganglia)	Brain	-2.5569064	0.34394907
chr20	2841437	2841438	C	T	ENSG00000215305	Brain - Putamen (basal ganglia)	Brain	-2.0199462	0.30552865
chr20	2841437	2841438	C	T	ENSG00000215305	Brain - Spinal cord (cervical c-1)	Brain	-2.5511674	0.34352627
chr20	2841437	2841438	C	T	ENSG00000215305	Brain - Substantia nigra	Brain	-2.3874208	0.33156925
chr20	49194968	49194969	C	T	ENSG00000196396	Brain - Caudate (basal ganglia)	Brain	-4.2331915	0.69278956
chr20	49194968	49194969	C	T	ENSG00000196396	Brain - Cerebellar Hemisphere	Brain	-4.816214	0.62332168
chr20	49194968	49194969	C	T	ENSG00000196396	Brain - Cortex	Brain	-4.4451761	0.66833238
chr20	49194968	49194969	C	T	ENSG00000196396	Brain - Frontal Cortex (BA9)	Brain	-4.7129842	0.63609962
chr20	49194968	49194969	C	T	ENSG00000196396	Brain - Hippocampus	Brain	-2.9986451	0.81284517
chr20	49194968	49194969	C	T	ENSG00000196396	Brain - Hypothalamus	Brain	-3.4355963	0.77497695
chr20	49194968	49194969	C	T	ENSG00000196396	Brain - Nucleus accumbens (basal ganglia)	Brain	-4.6301967	0.64621121
chr20	49194968	49194969	C	T	ENSG00000196396	Brain - Putamen (basal ganglia)	Brain	-3.7555046	0.74398667
chr20	49194968	49194969	C	T	ENSG00000196396	Brain - Spinal cord (cervical c-1)	Brain	-2.6484791	0.83949896
chr20	49194968	49194969	C	T	ENSG00000196396	Brain - Substantia nigra	Brain	-2.3446686	0.86006086
chrX	21887795	21887796	G	A	ENSG00000012174	Brain - Caudate (basal ganglia)	Brain	-0.5725068	0.6321225
chrX	21887795	21887796	G	A	ENSG00000012174	Brain - Cerebellar Hemisphere	Brain	-1.1403592	0.62390907
chrX	21887795	21887796	G	A	ENSG00000012174	Brain - Cortex	Brain	-0.6427706	0.63111024
chrX	21887795	21887796	G	A	ENSG00000012174	Brain - Frontal Cortex (BA9)	Brain	-0.4716242	0.63357384
chrX	21887795	21887796	G	A	ENSG00000012174	Brain - Hippocampus	Brain	-0.6355083	0.63121492

chrX	21887795	21887796	G	A	ENSG00000012174	Brain - Hypothalamus	Brain	-0.6427706	0.63111024
chrX	21887795	21887796	G	A	ENSG00000012174	Brain - Nucleus accumbens (basal ganglia)	Brain	-0.6427706	0.63111024
chrX	21887795	21887796	G	A	ENSG00000012174	Brain - Spinal cord (cervical c-1)	Brain	-0.574833	0.63208901
chrX	21887795	21887796	G	A	ENSG00000012174	Brain - Substantia nigra	Brain	-0.6427706	0.63111024

Supplementary Online Figures

Supplementary Figure 1 Validation of fibroblast RNA-seq results via proteomics

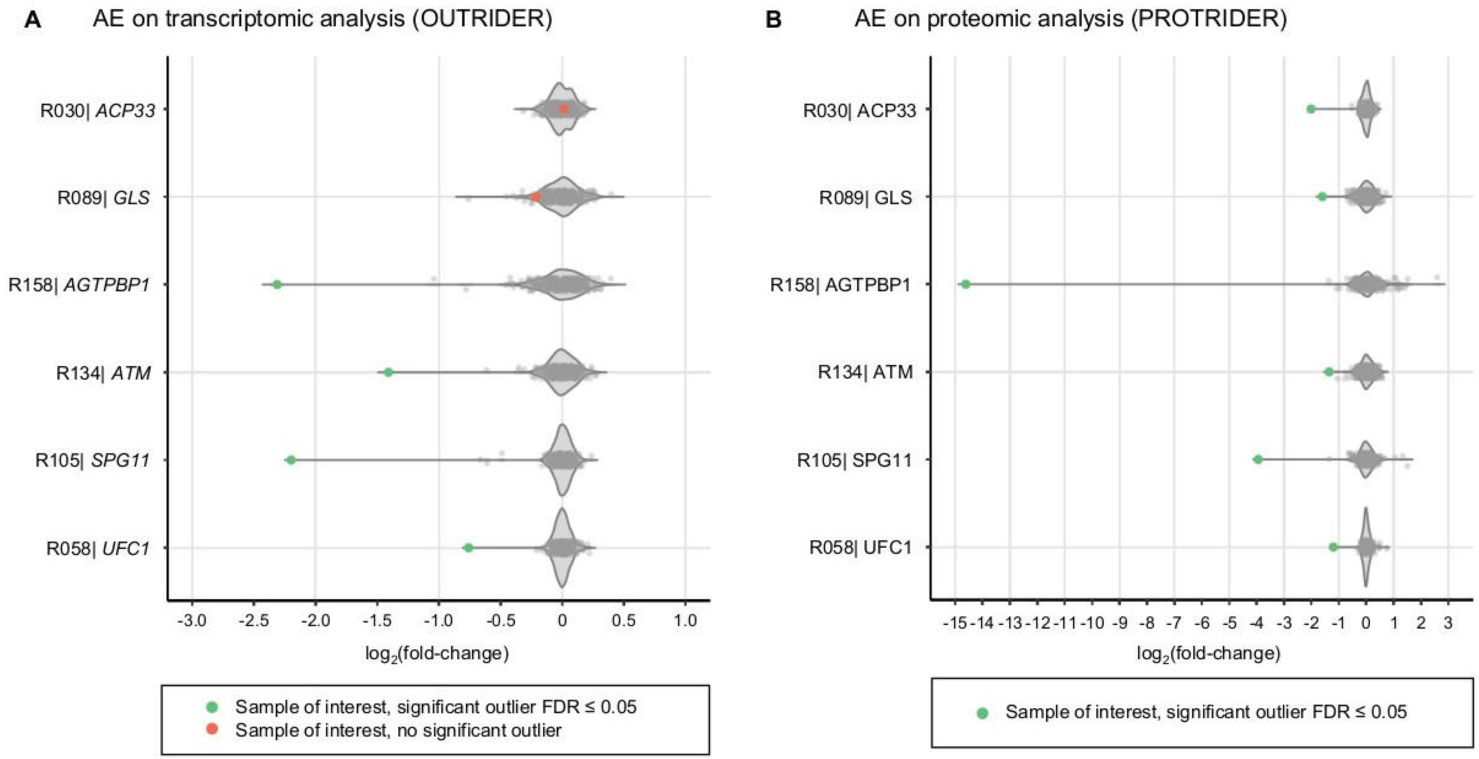

Quantitative proteomics analysis was performed on patient fibroblasts, as described (PMID: 39937650). Corresponding underexpression at the protein level was observed for patients with near-splice or deep(er) intronic causative variants in *ACP33*, *GLS*, *AGTPBP1*, *ATM*, *SPG11*, and *UFC1*, reported in **Table 1**.

Supplementary Figure 2 Validation of fibroblast RNA-seq results via Oxford Nanopore Technologies (ONT)-based long read RNA analysis

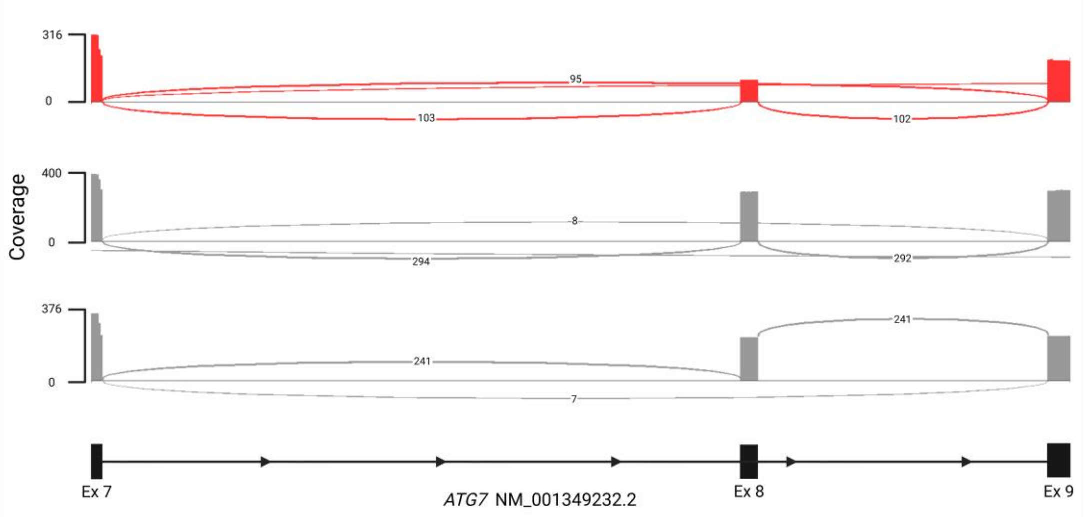

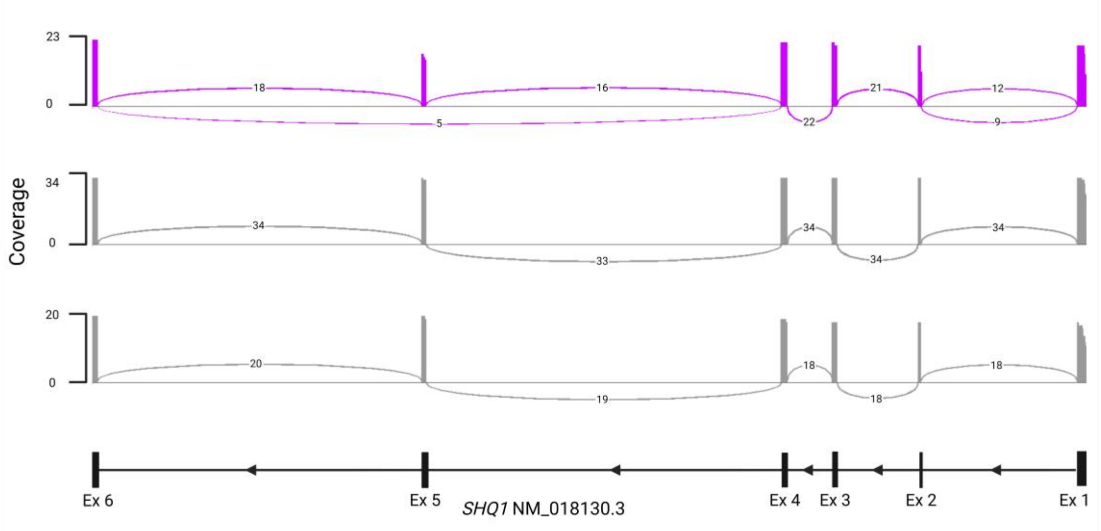

Long-read RNA analysis based on an Oxford Nanopore Technologies (ONT) platform was performed on patient fibroblasts to confirm the results from RNA-seq presented in this study for patient R054 (*ATG7* variant causing exon skipping, see **Table 1**; upper panel; R054 shown in red and two representative controls shown in gray) and patient R082 (*SHQ1* variants causing exon extension and exon skipping, see **Table 1**; bottom panel; R082 shown in purple and two representative controls shown in gray).

Supplementary Figure 3 Validation of fibroblast RNA-seq results via qPCR

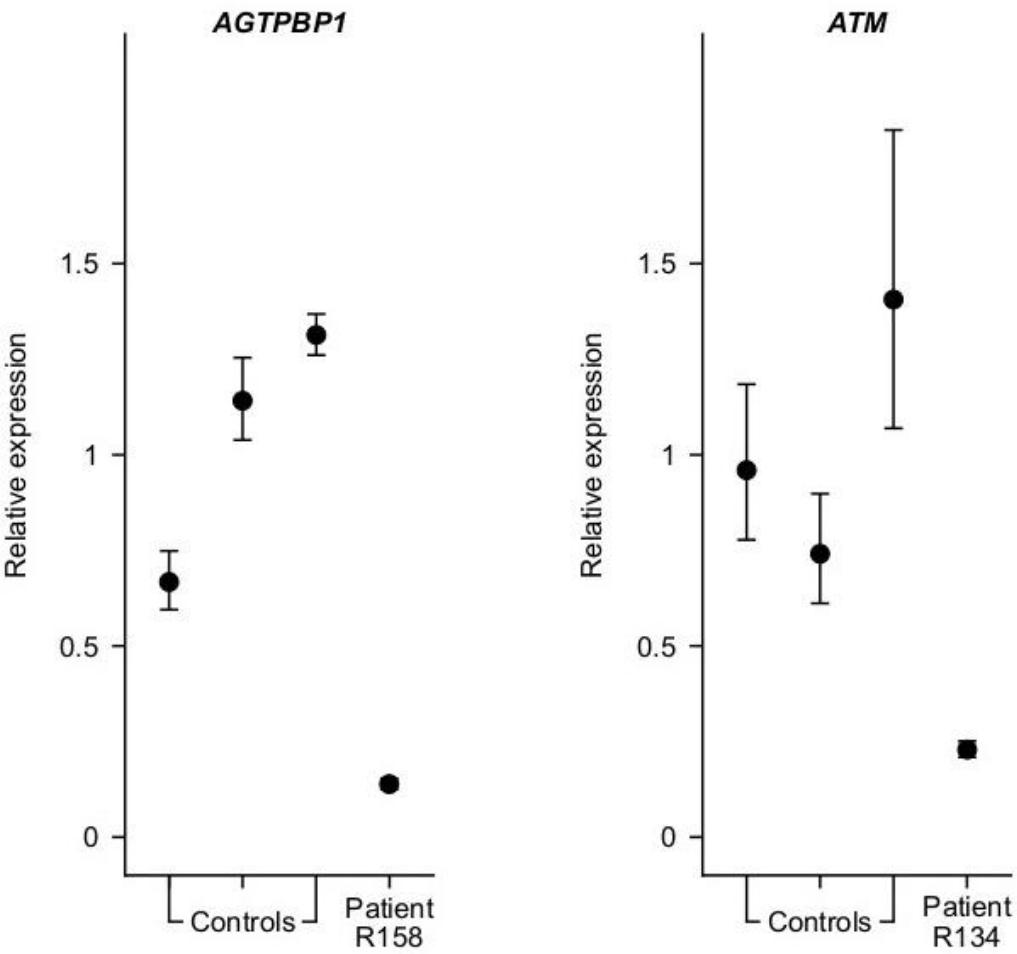

qPCR was performed on patient fibroblasts to confirm the results from RNA-seq presented in this study for patient R158 with *AGTPBP1* intronic variant causing *AGTPBP1* mRNA underexpression (see **Table 1**) and patient R134 with *ATM* intronic variants causing *ATM* mRNA underexpression (see **Table 1**).

Supplementary Figure 4 Predicted variant effects on RNA expression in the brain

A

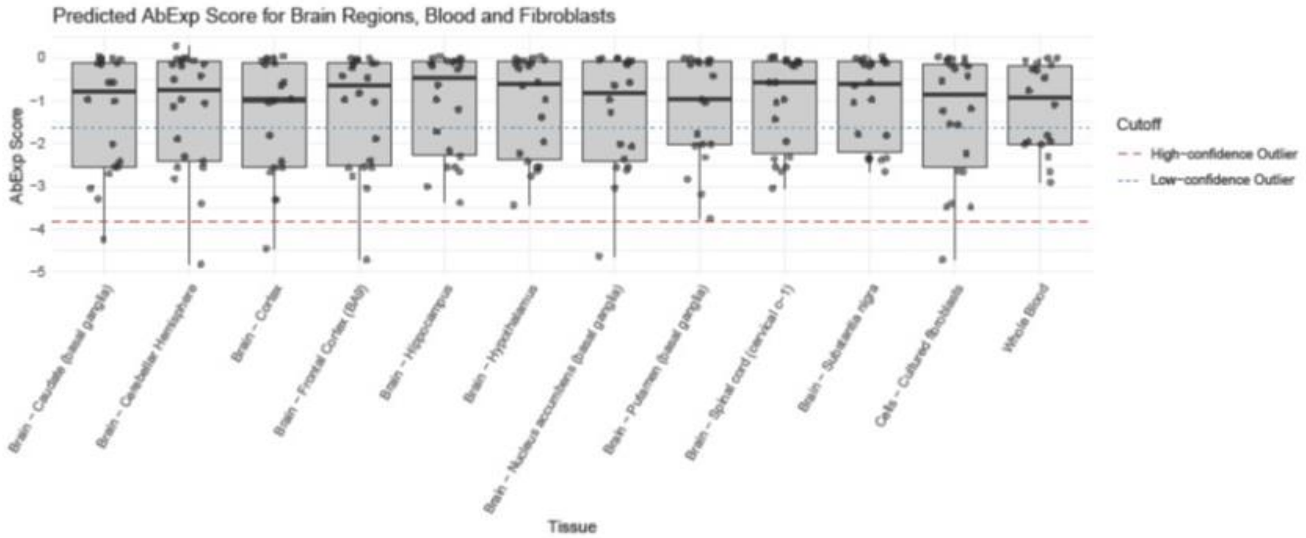

B

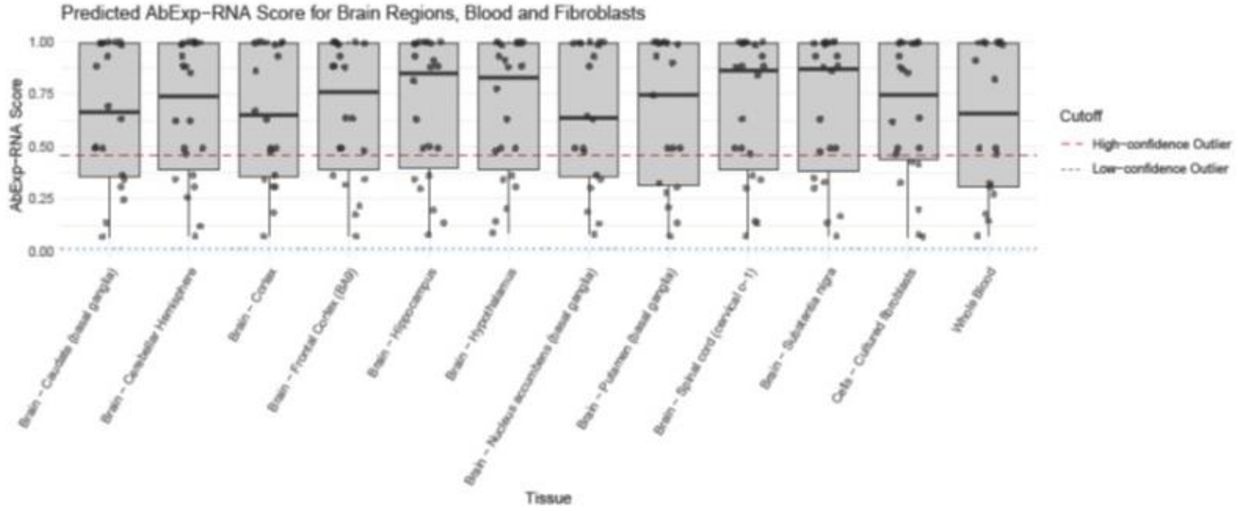

The machine-learning model AbExp was deployed to predict decreased RNA abundance as a result of herein-reported variants in different brain regions relevant to dystonia. Box plots for scores of gene expression are shown for the indicated brain regions. (A) AbExp predictions based on DNA-level data only. AbExp < -1.64 (blue dotted line) corresponds to low-confidence predicted underexpression outliers (20% precision, 21.5% recall); AbExp < -3.84 (red dotted line) corresponds to high-confidence predicted underexpression outliers (50% precision, 7.8% recall). (B) AbExp predictions based on DNA-level data plus fibroblast RNA-seq data. For this analysis, a logistic regression classifier was used to calculate the integrated DNA-based AbExp scores with outlier results using a binary indicator label of whether a gene is expressed in the clinically accessible tissue, the OUTRIDER z-score in the clinically accessible tissue, and the DNA-based AbExp score in target tissue, plus all three pairwise interaction terms, trained in cross-validation. AbExp-RNA > 0.013 (blue dotted line) corresponds to low-confidence predicted underexpression outliers (20% precision, 47.6% recall); AbExp-RNA > 0.459 (red dotted line) corresponds to high-confidence predicted underexpression outliers (50% precision, 22.8% recall). When incorporating RNA results into AbExp score predictions, we see improvements across all regions for outlier predictions, with a majority of outliers falling above the high confidence outlier cutoff. This is expected given that the selected variants were found in genes that were detected as true outliers in OUTRIDER. Additionally, the median predicted aberrant expression score in fibroblasts is now greater than the predicted blood score while the range is smaller, indicating that fibroblasts may serve as a more reliable surragate for expression prediction. However, both fibroblast and blood predicted AbExp scores aligns with predicted brain region scores indicating consistent predictions across different tissue types.

Supplementary Figure 5 Benchmarking of AbExp performance

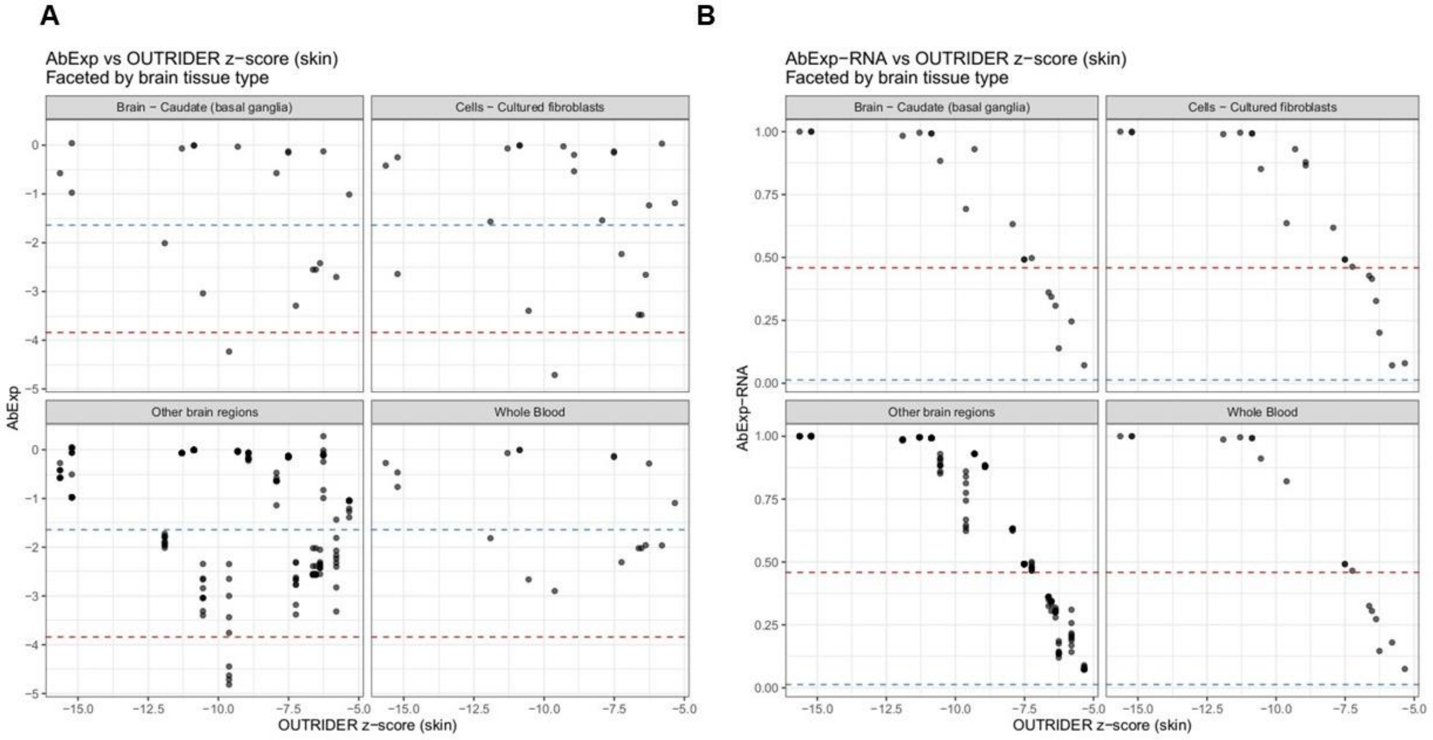

(A) When plotting AbExp score based on DNA variant information, one would expect a more extreme AbExp score (more negative) relative to a more extreme *z*-score from OUTRIDER. However, the correlation appears scattered indicating that the predicted expression is not well calibrated to the true expression seen in OUTRIDER. (B) When comparing the AbExp with RNA predicted scores against the calculated OUTRIDER *z*-scores from the fibroblast-based analysis, similar trends appear accross predicted Brain - Caudate (basal ganglia), other brain regions, whole blood, and cultured fibroblast cells: as the OUTRDIER *z*-score becomes more extreme, the AbExp predicted score increases as expected. All selected variants were known outliers from OUTRIDER which is supported by most predicted AbExp scores being above the high confidence cutoff and all being above the low confidence cutoff for outlier classification.
